# Open‐ and Closed‐Loop Recycling of Polyesters and Post‐Consumer Waste Under Industrially Relevant Conditions Using Bisguanidine Organocatalysts

**DOI:** 10.1002/cssc.202502062

**Published:** 2026-01-12

**Authors:** Lisa Burkart, Alisa Hahn, Damon Blum, Yasemin Kara, Alexander Hoffmann, Sonja Herres‐Pawlis

**Affiliations:** ^1^ Institute of Inorganic Chemistry RWTH Aachen University Aachen Germany

**Keywords:** alcoholysis, guanidine, organocatalysis, polyethylene terephthalate, polylactide recycling

## Abstract

With the increasing demand for sustainable plastic materials, the implementation of a circular plastics economy starting from designing environmentally friendly polymers and including effective recycling strategies is of the utmost importance. The biobased and biodegradable polyester polylactide (PLA) is a promising candidate for a sustainable, circular plastics economy. Polyesters can be chemically recycled to obtain monomers or value‐added chemicals following either a closed‐ or open‐loop recycling approach. However, the requirements for catalysts applicable in post‐consumer waste recycling are high: Besides a high activity, robustness and scalability of the catalyst are important factors. We studied highly active, robust bisguanidine organocatalysts for the depolymerization of the polyesters polycaprolactone, polyethylene terephthalate, and PLA. Focusing on PLA, we investigated the structure–reactivity relationship of the length of the aliphatic linker between the guanidine functionalities to identify the most active catalyst: Bis(*N*,*N*,*N′*,*N′*‐tetramethylguanidino)ethane (TMG_2_e) depolymerizes PLA completely *via* alcoholysis within minutes under mild reaction conditions. The bisguanidine shows excellent activity for the alcoholysis of the investigated polymers, the ability to depolymerize binary and ternary plastic mixes, and robustness against additives, plasticizers, and other impurities in different post‐consumer waste samples. Thus, TMG_2_e has promising properties to be an asset for the implementation of a sustainable, circular plastics economy.

## Graphical abstract

The chemical recycling of polylactide (PLA), polyethylene terephthalate (PET), and polycaprolactone (PCL) is performed using bisguanidine organocatalysts. Intensive kinetic studies of the methanolysis and ethanolysis of PLA were performed. The most active organocatalyst was successfully applied in the depolymerization of ternary plastic mixes of PLA, PCL, and PET as well as in the depolymerization of post‐consumer PET waste.

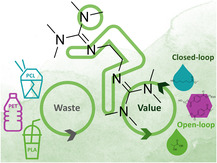

## 1 Introduction

**FIGURE 1:**
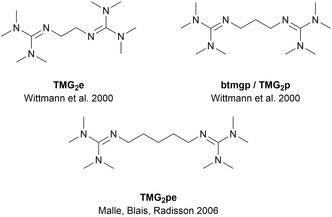
Investigated organocatalysts TMG_2_e [67], TMG_2_p [67, 68], and TMG_2_pe [69].

The increasing demand for plastics in various application fields is one of the most important challenges of the 21st century [1, 2]. Plastics are everywhere: from everyday household items and food and drink packaging to medical applications and spacecrafts [3, 4]. With increasing demand and increasing production comes an increasing amount of waste that needs to be managed [2, 5]; thus, establishing a sustainable, circular plastics economy is of utmost importance [6, 7, 8–9]. This goal can be achieved by enforcing the policies: reduce, reuse, recycle [2, 4, 10] and switching from petrol‐based to biobased polymers [3, 9]. One of the most promising bioplastics on the market is polylactide (PLA) [11]. PLA is derived from biomass and fully degradable to CO_2_ and water under industrial composting conditions [12, 13]. Alongside compostability, other recycling options that offer access to the monomer or new value‐added products are an important strategy in a circular economy. Effective recycling reduces the cost of monomer production, saves natural resources, and enables the creation of value from perceived waste material [14, 15, 16, 17, 18–19]. Besides mechanical recycling, which can lead to downcycling [15], chemical recycling is an efficient way to treat plastic‐rich waste. In a circular plastics economy, circularity is achieved *via* open‐ or closed‐loop recycling. Open‐loop recycling produces new value‐added products from end‐of‐life (EoL) plastics, whereas closed‐loop recycling can provide new monomers for polymerization of new polymers [20, 21, 22–23]. For PLA, both ways can be achieved using alcoholysis. The alcoholysis of PLA can be catalyzed by various metal complexes [21, 22, 24, 25, 26, 27, 28, 29, 30, 31, 32, 33, 34, 35, 36, 37, 38, 39, 40, 41, 42, 43, 44–46], organocatalysts [47, 48], and ionic liquids [17, 49, 50–51] under mild conditions [14–18, 51, 52, 53–55]. Hereby, the ester functionality in the PLA backbone is attacked by the alcohol used. The nucleophilic attack leads to scission of the polymer backbone *via* a transesterification reaction [25, 35, 36]. Depending on the conditions and the alcohol used, the alkyl lactate is formed either directly by scission of the polymer chain‐end (pathway A) or by random scission of the polymer backbone leading to the formation of oligomers (pathway B). The oligomers are then transformed into the alkyl lactate in an equilibrium reaction [20, 25, 28, 35, 36, 41]. Using metal catalysts, pathway A is observed in the absence of solvents and for reactions in solution using a specific nucleophile, that is, benzyl alcohol [33, 36]. Reactions in solution using simpler aliphatic alcohols follow pathway B [30, 33, 34, 36]. The obtained alkyl lactates can be used to regain the monomer lactide (LA) [56], other new monomers [45, 57], or serve as green solvents [42]. Besides working on the integration of biobased polymers such as PLA into a circular plastics economy, the obtained know‐how of chemical recycling of polyesters can be applied to conventional, mostly fossil‐based polyesters such as polyethylene terephthalate (PET). The mechanical recycling of PET bottles is one of the most prominent recycling approaches nowadays [3, 58, 59]. EoL PET from bottles can be collected in high purity enabling successful mechanical recycling to high‐quality PET [60, 61]. But, due to mismanagement of PET waste and its application in multilayer materials, other recycling approaches are necessary to valorize the whole potential of PET waste [58–60, 62]. The separation of waste material is difficult and for some processes, even small impurities can impact the quality of the product [3, 58, 59]. PLA and PET have similar properties, thus aggravating proper separation of mixed waste streams. They have similar densities and cannot be separated optically. For proper separation, near‐infrared spectrometers need to be applied but those are expensive, seldomly used in waste management facilities, and have limitations such as dark‐colored materials (e.g. black bottles). A solution to this problem could be chemical recycling [60, 63]. The alcoholysis is a promising chemical recycling route enabling proper separation of PET and PLA waste material, especially in a scenario in which further mechanical separation is not possible [63, 64–65]. Besides separation, working with waste materials entails a set of new challenges for chemical recycling: The applied processes and catalysts need to be robust against various impurities, such as organic waste and other polymers as well as plasticizers, pigments, fillers, and different additives [55, 61, 66]. In the last three years, we reported several guanidine metal catalysts active in the alcoholysis of PLA [29–34] and other polyesters such as PET, and polycaprolactone (PCL) [31, 34]. This study focuses on the introduction of bisguanidine organocatalysts as highly active and robust catalysts for post‐consumer waste depolymerization under industrially relevant conditions. We studied the easy‐to‐synthesize bisguanidines bis(*N*,*N*,*N′*,*N′*‐tetramethylguanidino)ethane (TMG_2_e) [67, 68], bis(*N*,*N*,*N′*,*N′*‐tetramethylguanidino)propane (TMG_2_p or btmgp) [67–68], and bis(*N*,*N*,*N′*,*N′*‐tetramethylguanidino)pentane (TMG_2_pe) [69] concerning their activity for the methanolysis and ethanolysis of PLA (Figure 1).

To investigate the influence of the CH_2_‐linker between the two guanidine functionalities, we determined the depolymerization rate constant *k*_dp_ for the methanolysis and ethanolysis for qualitative comparison with each other and other catalysts. Furthermore, the activation energy *E*_A_, the activation entropy Δ*S* ^≠^, and the activation enthalpy Δ*H* ^≠^ were determined. TMG_2_e showed the most promising properties; thus, we investigated the applicability of TMG_2_e under industrially relevant conditions, keeping the principles of Green Chemistry in mind [70, 71]. We performed the depolymerizations without solvents using unpurified chemicals [72]. The methanolysis and ethanolysis using TMG_2_e were scaled up to 50 g PLA (methanolysis) and 175 g PLA (ethanolysis). The obtained methyl lactate (MeLa) and ethyl lactate (EtLa) can either be used to regain the monomer LA [56] or as green solvents and food additives [73, 74]. TMG_2_e was further investigated in the depolymerization of other polyesters showing excellent results for the methanolysis of PCL. Furthermore, we performe the glycolysis and the methanolysis of pristine PET and post‐consumer PET waste material as well as the methanolysis of binary and ternary polymer mixes enabling the valorization of waste streams unfit for mechanical recycling.

## 2 Results and Discussion

### 2.1 Bisguanidines as Organocatalysts for the Alcoholysis of PLA

The recycling of post‐consumer waste plastic material under industrially relevant conditions requires catalysts that are highly active, robust, and easy to synthesize. Previously, Conrads et al. reported several easy‐to‐synthesize Fe hybrid [32, 33] and bisguanidine [32] complexes active in the LA ring‐opening polymerization and depolymerization of PLA. The Fe(II) catalysts [FeCl_2_(TMG_2_e)] and [FeCl_2_(TMG_2_p)] are synthesized using FeCl_2_ and the bisguanidines TMG_2_e and TMG_2_p [32, 67, 68]. Preliminary tests showed that TMG_2_e and TMG_2_p depolymerize PLA faster than the corresponding Fe complexes but have a lower selectivity for MeLa (*S*_MeLa_) at similar PLA conversions (Table S2). This observation indicates that the metal center, if present, has a significant impact on the alcoholysis of PLA: decreasing the conversion of the internal methine group of PLA (*X*_int_) and increasing the selectivity for the alkyl lactate (*S*_RLa_). We propose that the transition state of the metal catalyst is sterically more demanding, aggravating the polymer conversion. However, increasing the selectivity by favoring the more accessible polymer chain‐ends or smaller oligomers. Another difference to the Fe complexes is the robustness of the organocatalysts toward impurities like water or air. Furthermore, the bisguanidines can be synthesized in a one‐step reaction starting from the diamine and the tetramethylchloroformamidinium chloride Vilsmeier salt (TMG‐VS) [32, 33, 67, 68]. With the transfer to industrial application in mind, we synthesized TMG_2_e without protective atmosphere using technical‐grade solvents to cut the overall costs of the synthesis. TMG_2_e was obtained as a colorless solid in a good yield (71%) after sublimation at 135°C. This and the well‐established activity of guanidine functionalities for the depolymerization of polyesters make TMG_2_e and TMG_2_p promising candidates as organocatalysts active in the depolymerization of PLA [20, 47, 54].

To establish a baseline to evaluate the activity of the catalysts, we performed two control experiments without a catalyst: i) one under standard reaction conditions using MeOH (1.0 mL, 7.1 equiv) and PLA (film, 250 mg, 3.47 mmol, 1.00 equiv) at 60°C and at 260 rpm. After 22 h, no depolymerization of PLA could be observed using ^1^H NMR spectroscopic analysis (Table 1). ii) The second experiment was performed without tetrahydrofuran (THF) as solvent and with EtOH (1.4 mL, 24 mmol, 6.9 equiv) and PLA (powder, 250 mg, 3.47 mmol, 1.00 equiv) at 100°C and at 260 rpm (Section S6.2). Within 22 h, the polymer swelled, but did not dissolve. We observed traces of oligomeric species in the heterogeneous mixture using ^1^H NMR spectroscopic analysis. Thus, we propose that the observed conversion of PLA can be assigned to the catalysts used. First, we investigated if the CH_2_‐linker between the two guanidine functionalities has a beneficial effect compared to the single guanidine functionality of *N*,*N*,*N*′,*N*′‐tetramethyl guanidine (TMG). Table 1 shows the obtained results for the methanolysis (1.0 mL, 7.1 equiv MeOH) of PLA (250 mg, 3.47 mmol, 1.00 equiv) using standard conditions: 0.5–1.0 mol% TMG_2_e, TMG_2_p, and TMG_2_pe in THF forming a homogeneous solution (THF (4 mL)) at 60°C (stirring speed = 260 rpm). TMG was tested using 0.5 and 1.0 mol%. We doubled the catalyst loading of TMG to achieve the same number of guanidine functionalities present in the reaction mixture as for the bisguanidine catalysts. Table 1 shows the reaction time *t*, the *S*_RLa_, and the yield of the alkyl lactate (*Y*_RLa_) determined for a conversion *X*_int_ ≥ 98%. TMG_2_e, containing two CH_2_‐units, reached 98% after 10 min, whereas TMG_2_p required 15 min and TMG_2_pe required 20 min. TMG (1 mol%) shows a lower apparent reaction rate constant *k*_app_ than the bridged bisguanidine catalysts and reaches *X*_int_ = 98% after 3 h. 0.5 mol% of TMG showed an even lower conversion of PLA as expected. Thus, we propose a structure‐reactivity relationship between the length of the linker and the reactivity of the catalysts. Our results indicate a beneficial influence of a linker on the alcoholysis and an increased reactivity for shorter linkers. We propose that both guanidine functionalities are involved in the scission of the polymer backbone (Scheme S1). *S*_MeLa_ is low until complete conversion is achieved and increases with time as MeLa is formed *via* an equilibrium reaction between the oligomers and MeOH (Figure S1).

**TABLE 1:** Overview of obtained *X*_int_, *S*_MeLa_, *Y*_MeLa_, and *k*_app_ for the methanolysis (7.1 equiv MeOH) of PLA (250 mg, 3.47 mmol, 1.00 equiv) using TMG_2_e, TMG_2_p, TMG_2_pe, TMG, and no catalyst in THF (4 mL) at 60°C and 260 rpm.

Catalyst	# CH_2_ a	cat. loading^b^ (mol%)	*t* (min)	*X*_*int*_ c (%)	*S*_MeLa_ c (%)	*Y*_MeLa_ c (%)	*k*_app_ (min^−1^)
TMG_2_e	2	0.5	10	98	46	45	0.346 ± 0.002
TMG_2_e	2	1.0	3	99	51	51	n.d.
			10	100	91	91	
TMG_2_p	3	0.5	15	99	51	50	0.217 ± 0.003
TMG_2_p	3	1.0	5	98	59	59	n.d.
			25	100	98	98	
TMG_2_pe	5	0.5	20	100	61	61	0.274 ± 0.016
TMG	–	0.5	180	66	19	12	0.00629 ± 0.00018
TMG	–	1.0	180	98	40	39	n.d.
‐d	–	–	1320	0	0	0	n.d.

a Number of CH_2_ groups of the linker between the guanidine functionalities.

b Catalyst loading in regards to ester bonds in the PLA used.

c *X*_int_, *S*_RLa_, and *Y*_RLa_ were calculated from ^1^H NMR spectroscopic analysis according to literature [28].

d No catalyst was used.

In comparison with other organocatalysts active in the alcoholysis of PLA, all three bisguanidine catalysts show promising results [47, 54]. McKeown et al*.* introduced the cheap organocatalyst [NMe_3_]^+^[OCO_2_Me]^−^ able to depolymerize PLA within 1 h using various solvents at 50°C [17]. In THF, *Y*_MeLa _= 83% could be reached within 1 h using 0.5 mol% of catalyst [17]. In 2011, 1,5,7‐triazabicyclo [4.4.0]dec‐5‐ene (TBD) was introduced as a highly active, versatile catalyst for the alcoholysis of PLA using different alcohols [20]. Complete methanolysis of PLA could be observed within 2 min in methylene chloride at room temperature [20]. TBD has one guanidine functionality but shows a fast conversion of PLA under the reported reaction conditions. Another highly active organocatalyst for the alcoholysis of PLA was introduced by Alberti et al. They reported 4‐dimethylaminopyridine (DMAP) active in the microwave‐assisted methanolysis of PLA without solvent at 180°C [47]. Due to the varying reaction conditions, a direct comparison between these literature‐known catalysts and the three investigated bisguanidine catalysts is difficult. However, TMG_2_e, TMG_2_p, and TMG_2_pe achieve complete conversion of PLA within short time periods under very mild reaction conditions (Table 1). TMG_2_e (1 mol%) depolymerizes PLA within 3 min completely and we achieve high yields of MeLa (*Y*_MeLa_ = 91%) within 10 min. TMG_2_p (1 mol%) shows similar results after 5 min (*X*_PLA_ = 98%) and reaches *Y*_MeLa _= 98% within 25 min. To gain a better understanding of the activity of the bisguanidines, the influence of the two guanidine functions, and the length of the linker between them, we investigated the methanolysis and ethanolysis of PLA. Therefore, we determined *k*_dp_ of the first depolymerization step and evaluated the catalysts concerning their *E*_A_, Δ*S* ^≠^, and Δ*H* ^≠^.

### 2.2 Evaluation of *k*_dp_

For the determination of *k*_dp_, kinetic experiments were performed using different catalyst loadings under standard reaction conditions (250 mg PLA, 7 equiv alcohol, 4 mL THF, 60°C, 260 rpm) for each catalyst and alcohol. The *k*_app_ values (Figures S5–S9) were determined and plotted against the catalyst loading to obtain *k*_dp_.

**FIGURE 2:**
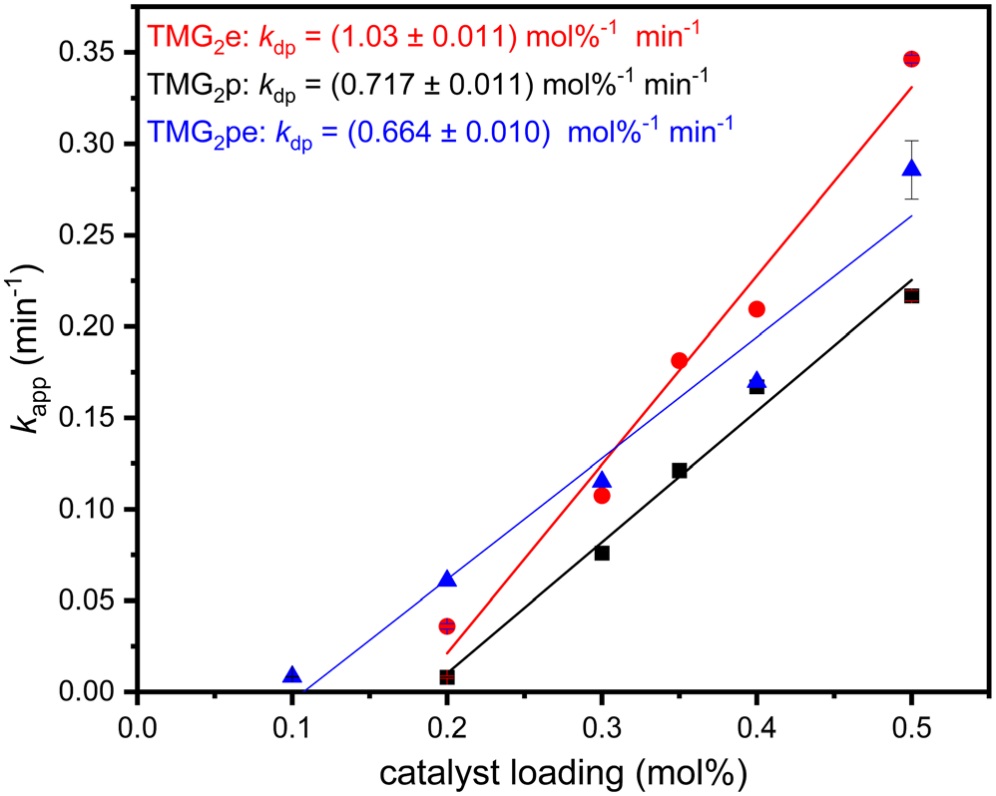
Plot of *k*_app_ against the catalyst loading in mol% for the methanolysis of PLA (250 mg, 3.47 mmol, 1.00 equiv) using TMG_2_e (red dots), TMG_2_p (black squares), and TMG_2_pe (blue triangles) using MeOH (1.0 mL, 7.1 equiv) in THF (4 mL) at 60°C and 260 rpm. All obtained linear regressions show signs of catalyst deactivation as they are no lines through the origin.

**FIGURE 3:**
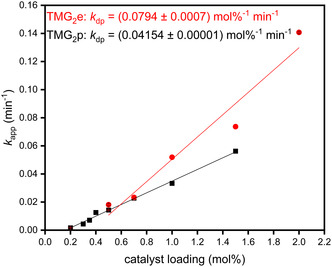
Plot of *k*_app_ against the catalyst loading in mol% for the ethanolysis of PLA (250 mg, 3.47 mmol, 1.00 equiv) using TMG_2_e (red dots) and TMG_2_p (black squares) using EtOH (1.4 mL, 6.9 equiv) in THF (4 mL) at 60°C and 260 rpm. All obtained linear regressions show signs of catalyst deactivation as they are no lines through the origin.

Figure 2 displays the obtained *k*_dp_ values for the methanolysis of PLA using TMG_2_e (red, (1.03 ± 0.011) mol%^−1^ min^−1^), TMG_2_p (black, (0.717 ± 0.011) mol%^−1^ min^−1^), and TMG_2_pe (blue, (0.664 ± 0.010) mol%^−1^ min^−1^). The linear regressions for the obtained *k*_dp_ values cut the y‐axis below zero. This behavior can be explained by catalyst deactivation under the given reaction conditions. The obtained *k*_dp_ values show that with the increasing length of the CH_2_‐linker, the activity for the methanolysis decreases further undermining the beneficial influence of a short linker. In Figure 3, a similar trend is displayed for the ethanolysis: The obtained *k*_dp_ for the ethanolysis using TMG_2_e (red, (0.0794 ± 0.0007) mol%^−1^ min^−1^) is higher than using TMG_2_p (black, (0.04154 ± 0.00001) mol%^−1^ min^−1^). As reported previously, we observed a significant influence of the alcohol on the reaction progress [30, 33, 34, 42]: The *k*_dp_ value for the ethanolysis using TMG_2_e and TMG_2_p is reduced by one order of magnitude compared to the methanolysis of PLA. This decrease in activity switching from MeOH to EtOH can be observed using various catalysts [20, 31, 33, 34, 42, 75]. Petrus et al. discuss the influence of the number of C atoms of the alcohols used and propose a beneficial impact of shorter alcohols with high nucleophilicity. [75] Further investigations of the alcoholysis using more challenging alcohols could provide more insight into their influence on TMG_2_e and TMG_2_p.

Compared to literature‐known metal catalysts, all three bisguanidine organocatalysts exhibit higher activity for the methanolysis and the ethanolysis of PLA in THF [30, 33, 34]. Very recently, we reported the bisguanidine Zn complex [Zn(DMEG_2_ch)_2_](OTf)_2_ · THF as a promising catalyst for the alcoholysis of PLA with a *k*_dp _= (0.078 ± 0.001) mol%^−1^ min^−1^ for the methanolysis and a *k*_dp _= (0.028 ± 0.001) mol%^−1^ min^−1^ for the ethanolysis [34]. The bisguanidine Zn catalyst shows a higher activity for the methanolysis of PLA than the hybrid guanidine Zn catalyst [ZnCl_2_(TMGeech)] [31] as well as the hybrid guanidine Fe catalyst [FeCl_2_(TMG5NMe_2_asme)] [33]. The determined *k*_dp_ values for all three bisguanidine organocatalysts are higher than for the bisguanidine Zn catalyst: TMG_2_e has a 13‐fold higher *k*_dp_ value for the methanolysis and 25‐fold higher *k*_dp_ value for the ethanolysis [34]. The use of organocatalysts increases the activity whilst reducing the number of steps and the overall costs of the catalyst synthesis facilitating the translation of the processes from laboratory to industry.

### 2.3 Evaluation of *E*_A_, Δ*S* ^≠^, and Δ*H* ^≠^

**FIGURE 4:**
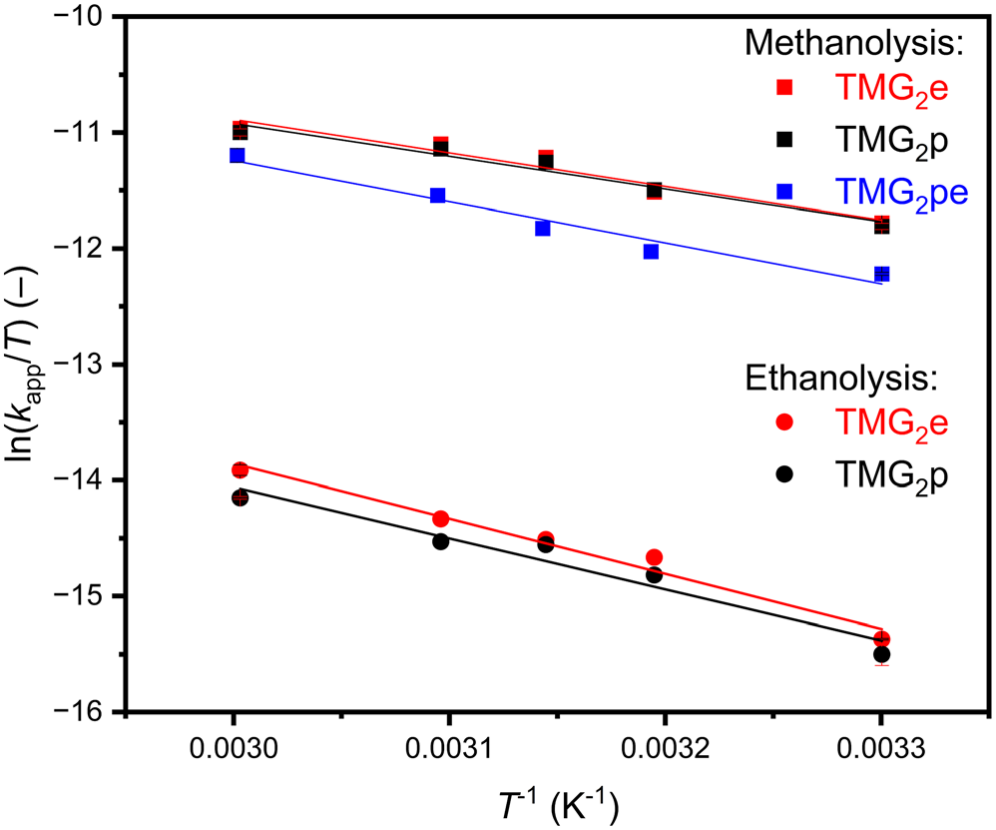
Eyring plots for the methanolysis (7.1 equiv, squares) and ethanolysis (6.9 equiv, dots) of PLA (250 mg, 3.47 mmol, 1.00 equiv) using 0.5 mol% of TMG_2_e (red), TMG_2_p (black), and TMG_2_pe (blue) in THF (4 mL) at 30, 40, 45, 50, and 60°C at 260 rpm.

To gain insight into the temperature dependency of the alcoholysis, experiments under standard reaction conditions (250 mg (3.47 mmol, 1.00 equiv) PLA, 0.5 mol% catalyst, 7 equiv alcohol, 4 mL THF, 260 rpm) were performed at 30, 40, 45, 50, and 60°C. In Figure 4, the obtained Eyring plots for the methanolysis (squares) and the ethanolysis (dots) show a similar linear regression for TMG_2_e and TMG_2_p, whereas the C_5_‐linker of TMG_2_pe has a significant impact on the results for *E*_A_, Δ*S* ^≠^, and Δ*H* ^≠^. The obtained Arrhenius plots for the methanolysis and ethanolysis are shown in Figure S10. Here, similar behavior of the obtained plots can be observed. Table 2 lists the calculated values for *E*_A_, Δ*S* ^≠^, and Δ*H* ^≠^. TMG_2_e and TMG_2_p have the same values for all investigated parameters regardless of the alcohol. Therefore, the elongation of the linker by one CH_2_ unit seems to have no influence on the temperature dependency.

**TABLE 2:** Overview of obtained *E*_A_, Δ*S* ^≠^, and Δ*H* ^≠^ for the methanolysis (7.1 equiv MeOH) and ethanolysis (6.9 equiv EtOH) of PLA (250 mg, 3.47 mmol, 1.00 equiv) using 0.5 mol% catalysts in THF (4 mL) at 30, 40, 45, 50, and 60°C and 260 rpm.

Catalyst	#CH_2_ a	ROH	*E*_A_ (kJ mol^−1^)	Δ*H* ^≠^ (kJ mol^−1^)	Δ*S* ^≠^ (J mol^−1^ K^−1^)
TMG_2_e	2	MeOH	26.7 ± 0.4	24.1 ± 0.4	−216 ± 1
TMG_2_p	3	MeOH	26 ± 1	23 ± 1	−218 ± 5
TMG_2_pe	5	MeOH	32 ± 2	30 ± 2	−202 ± 6
TMG_2_e	2	EtOH	42 ± 1	40 ± 1	−194 ± 4
TMG_2_p	3	EtOH	39 ± 3	37 ± 3	−205 ± 9

a Number of CH_2_ groups of the linker between the guanidine functionalities.

The C_2_‐ and C_3_‐bridged organocatalysts show lower values for *E*_A_ and Δ*H* ^≠^ than Fe hybrid guanidine [33] and Zn bisguanidine complexes [34] explaining the faster conversion of PLA under comparable reaction conditions (see also Table 1). The values for *E*_A_ and Δ*H* ^≠^ for the methanolysis of the C_5_‐bridged TMG_2_pe are similar to the values determined for the Zn catalyst [Zn(DMEG_2_ch)_2_](OTf)_2_ · THF (*E*_A _= 36 ± 2 kJ mol^−1^; Δ*H* ^≠ ^= 33 ± 2 kJ mol^−1^) presented by Becker et al. in 2024. However, TMG_2_pe depolymerizes the methanolysis faster than the metal complex. All organocatalysts show a more negative Δ*S* ^≠^ than reported metal complexes [33, 34], which suggests an associative reaction mechanism. Comparing the results of TMG_2_p and TMG_2_e for the methanolysis with the results for the ethanolysis exhibits a similar trend as reported in literature [34, 42]. The methanolysis has a lower *E*_A_ and Δ*H* ^≠^, thus facilitating the conversion of PLA.

### 2.4 Mechanistic Consideration

**SCHEME 1:**
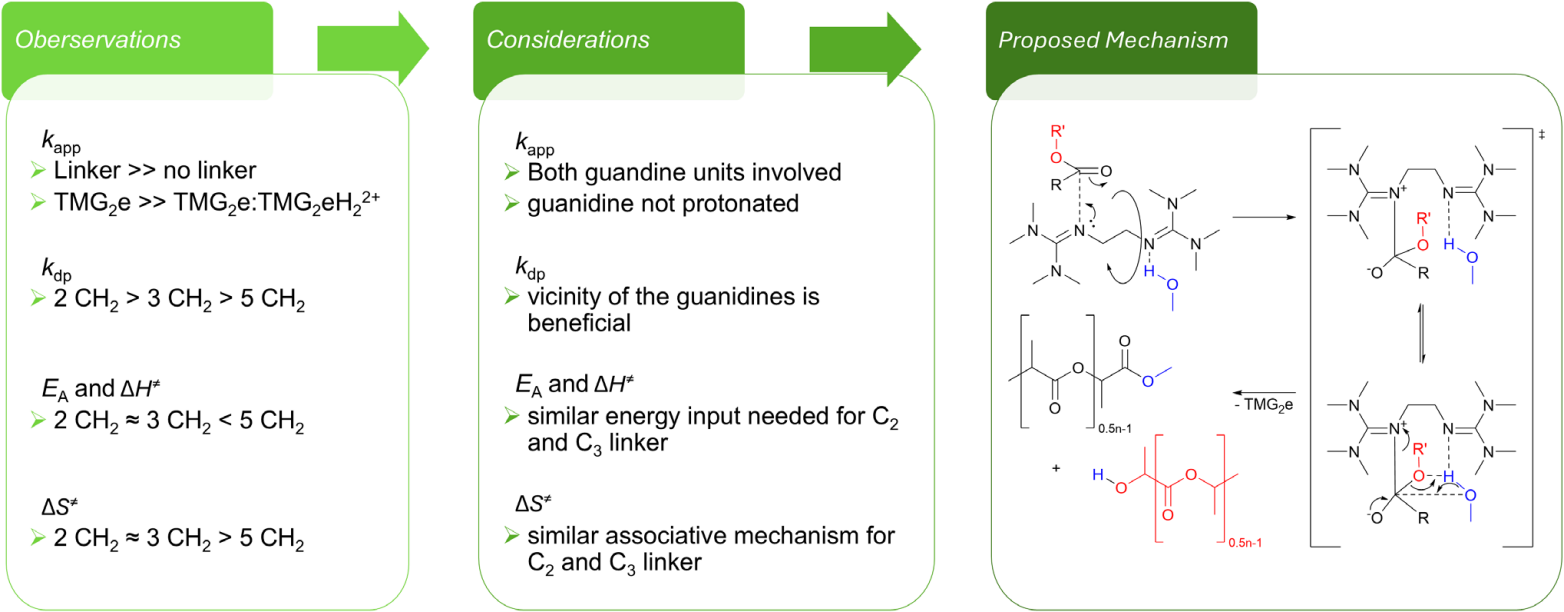
Proposed dual nucleophilic activation mechanisms for the methanolysis of PLA using TMG_2_e. The chain scission leads to the formation of two oligomers with different chain ends.

To gain insight into the mechanism of the alcoholysis using bisguanidine organocatalysts, we drew conclusions from the performed experiments and performed further experiments to exclude other theories. The presented results suggest that the length of the linker between the guanidine functionalities influences the alcoholysis. The elongation of the linker from two to three CH_2_ units has no significant impact on the determined *E*_A_, Δ*H* ^≠^, and Δ*S* ^≠^; whereas, an elongation of the linker to five CH_2_ units leads to higher *E*_A_ and Δ*H* ^≠^, and a less negative Δ*S* ^≠^. However, the shorter linker of TMG_2_e significantly improves the *k*_dp_ value compared to TMG_2_p and TMG_2_pe. Thus, we observe a beneficial effect of the spatial proximity of the guanidine functionalities. A similar trend was recently reported by Kamaraj et al*.* for the polymerization of LA to PLA using multiple bis‐ and monoguanidine organocatalysts [76]. The presented kinetic experiments suggest that the methanolysis and ethanolysis using all three bisguanidine organocatalysts follow a pseudo‐first‐order mechanism; therefore, only one catalyst molecule plays a role in the scission of the polymer backbone. Furthermore, the significant increase in the activity of the bisguanidines compared to TMG (Table 1) suggests that both guanidine functionalities are involved in the backbone scission of PLA. To further investigate the mechanism of the methanolysis using TMG_2_e, we synthesized the hydrochloride of TMG_2_e (TMG_2_eH_2_Cl_2_) [67]. The methanolysis using the hydrochloride of TMG_2_e showed complete inhibition of the catalysis. To further investigate the negative effect of the protonation of the catalyst, an experiment using a 1:1 ratio of TMG_2_e (1 mol%) to the hydrochloride was performed (Table S4). The obtained *k*_app_ (0.0637 min^−1^) is a magnitude lower than the obtained *k*_app_ value for 0.5 mol% TMG_2_e (Table 1). Nonetheless, the *k*_app_ obtained for the methanolysis of PLA using the 1:1 catalyst mixture is still high compared to other catalysts under similar conditions and shows the robustness of TMG_2_e against additional protons [32–34, 46]. Additionally, MeOH does not protonate the guanidine functionalities. These observations dismiss a transition state in which a protonated guanidine function activates the carbonyl oxygen in the polymer backbone as proposed for TBD [20]. Instead, we propose a dual nucleophilic activation mechanism *via* the imines of the two guanidine functionalities as displayed in Scheme 1 [20, 77, 78, 79–80]: One guanidine functionality could activate the electrophilic carbonyl C atom of the polymer backbone, the other could activate MeOH *via* an H‐bond.

We suggest that the higher activity for the ethylene‐bridged TMG_2_e routes in the spatial proximity of the guanidine functionalities and the fewer possibilities to arrange the reactive partners, thus facilitating the chain scission by aligning the polymer backbone with the alcohol more efficiently. The proposed mechanism is plausible for TMG_2_p. For TMG_2_pe, the mechanism might be aggravated due to the C_5_‐linker explaining the higher *E*_A_ and Δ*H* ^≠^. The longer chain could lead to a less simultaneous reaction process (less negative Δ*S* ^≠^), in which the alcohol functionality of the polymer (OR′) functions as a leaving group prior to the attack of MeOH. This could lead to a reversible protonation of the catalyst by the proton of MeOH or longer coordination of the ester chain end to the catalyst. Protic impurities such as water could hinder the reaction by protonating the OR′‐functionality of the polymer themselves, which might lead to protonation of the catalyst during the transition state with the proton from MeOH, and hence, reducing the catalytic activity.

### 2.5 Industrial Relevance

Real‐life applications entail new challenges such as the separation of plastic mixes and handling materials with varying compositions. Catalysts used for industrial applications need to be robust toward impurities such as water and air as well as toward other plastics and additives. Further, the catalysts need to be able to handle feedstocks with varying quality and composition. This is even more important considering mixed plastic waste streams. The reaction conditions should be as mild as possible while still achieving an effective catalysis and reducing costs and environmental impact. With these challenges in mind, TMG_2_e was investigated under industrially relevant conditions: We performed experiments in contact with air and used chemicals as bought. The ethanolysis and methanolysis were performed without solvent to reduce the amount of chemicals used. Further, we investigated the challenges of depolymerizing binary and ternary plastic mixes of PLA, PCL, and PET, and performed multiple depolymerization reactions using post‐consumer waste materials provided by Vestforbrænding (Denmark) and AIMPLAS (Spain).

### 2.6 Large‐Scale Methanolysis and Ethanolysis

The large‐scale methanolysis (5–50 g of PLA) and ethanolysis (5–175 g of PLA) were performed at 60, 100, and 150°C. Preliminary small‐scale methanolysis (7.1 equiv) of PLA (250 mg, 3.47 mmol, 1.00 equiv) using 1 mol% of TMG_2_e under solvent‐free conditions at 150°C achieved complete selective depolymerization of PLA to MeLa (*Y*_MeLa _= 98%) within 5 min. Thus, to reduce the used chemicals, and to facilitate the purification process of the obtained alkyl lactates, we performed the reactions without THF. At temperatures below 150°C, the methanolysis and ethanolysis are heterogeneous reactions; neither EtOH nor MeOH acts as solvent for PLA [73]. Thus, we were not able to monitor the reactions as closely using ^1^H NMR spectroscopic analysis as the reactions using THF as solvent. In Table S6, an overview of the obtained *X*_int_, *S*_RLa_, and *Y*_RLa_ for the depolymerizations is given after a homogenous reaction mixture was observed. Moreover, we reduced the amount of alcohol used from 7 equiv down to 3.7 equiv (120 mL EtOH). Here, the limiting factor is the stirring behavior if less alcohol is used. To further reduce the overall input of reagents, we collected the alcohol removed during the isolation process of EtLa and reused it successfully in another ethanolysis (Table 3). The methanolysis was scaled up to 50 g PLA and the ethanolysis was scaled up even further to 175 g PLA due to ethanol being the less toxic, biobased alcohol.

**TABLE 3:** Large‐scale methanolysis and ethanolysis of PLA using 1 mol% TMG_2_e without THF as solvent.

ROH	*m*_PLA_ (g)	*V*_ROH_ (mL)	*T* (°C)	*t* (h)	*Y*_RLa_ a (%)	*Y*_isolated_ (%)
MeOH	5	20	150	0.25	100	–
MeOH	50	200	60	1.25	100	54
EtOH	5	29	60	23	100	76
EtOH^b^	175	750	60–100	2 weeks	–	71
EtOH	30	170^c^	100	24	89	65
EtOH	40	120	100	6	–	82

a Determined with ^1^H NMR spectroscopic analysis and calculated according to literature [28].

b 0.5 mol% TMG_2_e.

c Recycled EtOH from the ethanolysis of 175 g PLA was used.

First, we tested a smaller approach using 5 g of PLA reaching complete selective methanolysis after 15 min. For the isolation of MeLa, the reaction approach was scaled up to 50 g. The reaction was conducted at 60°C to reduce energy consumption. After 1.25 h, ^1^H NMR spectroscopic analysis showed complete conversion of PLA to MeLa. The product could be isolated in a moderate yield of 54% using rotary evaporation. The relatively low isolated yield is explained by the evaporation of MeLa during the lab‐scale purification process. A technical distillation process could improve the isolation of MeLa and increase the yield significantly. The ethanolysis was performed using 5 g of PLA under mild reaction conditions (60°C). Full conversion to EtLa was observed after 23 h using ^1^H NMR spectroscopic analysis. We isolated EtLa in higher yields and purity (99%) compared to MeLa using the same purification procedure. The scale‐up to 175 g PLA in a 2 L flask was performed using 0.5 mol% catalyst and a reaction temperature of 60°C. High conversion to EtLa could be detected directly from the reaction mixture using ^1^H NMR spectroscopy after 20 h (*Y*_EtLa _= 72%), but PLA and oligomers were still present after 8 days under these reaction conditions. After 8 days, the reaction was heated to reflux conditions (*T*_oil bath _= 100°C) to accelerate the conversion of PLA. After an additional 25 h, PLA was depolymerized completely and EtLa was isolated in a good yield. The alcohol removed during the isolation process was reused in the ethanolysis using 30 g of PLA (Table 3). We applied optimized reaction conditions (reflux conditions, 1 mol% TMG_2_e) and isolated EtLa (*Y*_EtLa_ = 65%) in high purity (>99%). To further minimize the amount of used chemicals, we reduced the amount EtOH to 3.7 equiv in regards to PLA (40 g). Using 1 mol% of TMG_2_e, the heterogeneous reaction mixture was homogenized within 2 h due to complete depolymerization of PLA (*X*_int_ = 100%, *Y*_EtLa_ = 89%). To achieve a higher selectivity, the reaction was treated to reflux conditions for additional 4 h, and we could isolate EtLa in good yields (*Y*_EtLa_ = 82%). These results suggest that the alcoholysis of PLA using TMG_2_e under mild reaction conditions is easily scalable and adjustable to specific needs.

### 2.7 Depolymerization of PCL and PET

Besides PLA, the activity of TMG_2_e toward the depolymerization of fossil‐based polyesters in contact with air and under solvent‐free conditions was investigated. PET can be depolymerized to dimethyl terephthalate (DMT) using MeOH and bis(2‐hydroxyethyl)terephthalate (BHET) using ethylene glycol (EG) [14, 17, 31, 34]. Both depolymerization products can be used to produce PET [28, 81, 82]. PCL was depolymerized to methyl 6‐hydroxyhexanoate (MeHe) using MeOH. MeHe serves as a monomer to produce PCL [22, 31, 34]. Table 4 shows the applied reaction conditions and the obtained yields of the corresponding depolymerization product (*Y*_dp_). PCL was depolymerized at 100°C to MeHe within 60 min (*Y*_dp_ = 97%). TMG_2_e shows fast depolymerization of PCL under mild reaction conditions compared to literature‐known guanidine Zn catalysts [31, 34]. For example, Cheung et al. reported that Zn(OAc)_2_ (2 mol%) depolymerizes PCL at elevated temperatures of 140–170°C achieving a yield of 92% within 60 min [22].

**TABLE 4:** Results for the solvent‐free depolymerization of PET (3.5 mmol) and PCL (3.4–3.5 mmol) using 1 mol% TMG_2_e with regards to ester bonds using different nucleophiles (Nu: MeOH = 4 mL and EG = 4 mL).

Polymer	*m*_polymer_ (g)	Nucleophile	*T* (°C)	*t* (h)	*Y*_dp_ (%)^a^
PCL^b^	0.40	MeOH	100	60	97 ± 0^c^
PET	0.67	MeOH	180	5	90
PET	0.67	EG	180	24	61
PET	3.0	EG	180	30	76
PET [84]	5.0	EG	180	6 days	61

a Isolated yield.

b 2.0 mol% TMG_2_e.

c Determined with ^1^H NMR spectroscopic analysis and calculated according to literature [22].

The methanolysis of PET yielded DMT in high isolated yields (*Y*_DMT_ = 90%) compared to Zn catalysts under similar reaction conditions (150°C, 6 h, *Y*_DMT_ = 65%) [17, 34]. The organocatalyst [NMe_3_]^+^[OCO_2_Me]^−^ achieved comparable yields of DMT within 16 h at lower temperatures but with a higher catalyst loading [17]. Another commonly used depolymerization method of PET is the glycolysis [17, 54, 82, 83]. Table 4 shows the results of the glycolysis of PET using TMG_2_e. We were able to scale up the reaction from 0.67 g scale to 5.0 g scale with high isolated yields (*Y*_BHET_ = 61–76%) under solvent‐free conditions [84]. During the glycolysis, the reaction mixture turned dark brown, which led to brown coloration of the obtained BHET, which was achieved as a brown solid. The methanolysis of PET under similar conditions shows no discoloration. This indicates that under these conditions, the catalyst is stable. A control reaction using only EG and TMG_2_e led to no signs of the formation of polyethylene glycol and the reaction mixture remained colorless. To further investigate the color change, we performed three control experiments using the BHET obtained from the 5 g scale‐up experiment. First, BHET and THF were heated to 180°C and stirred for 5 h at 260 rpm. The reaction mixture turned from light brown to dark brown and ^1^H NMR spectroscopic analysis showed decomposition of BHET. In a second and third experiment, BHET and TMG_2_e were dispersed in THF or EG, heated to 180°C, and stirred for 5 h at 260 rpm. The mixture in THF turned from light brown to dark brown within 1 h and showed more decomposition of BHET than the experiment using EG: Using EG, the reaction mixture turned dark brown within 3 h. Contrary to the other reactions in THF, here, BHET can still be identified as the main species. These results suggest that under the given reaction conditions, BHET is prone to decompose. Thus, milder reaction temperatures could lead to fewer side reactions and improve the color of the product. In this study, we were able to improve the discoloration by washing the product with deionized water. The obtained light gray BHET from the 5 g scale‐up experiment using pristine PET, listed in Table 4, and the 7 g scale‐up experiment using PET trays, listed in Table 5, were applied successfully in the production of PET. In a previously published study, BHET was polymerized to PET using microwave‐assisted solid‐state polymerization [84]. The polycondensation of BHET was performed in three heating cycles with Sb_2_O_3_ and Ti(OBu)_4_ ([catalyst]:[BHET] = 0.0001) as catalysts. The performed experiments produced a low molecular weight PET with increased discoloration due to the coloration of the BHET produced in this study [84]. To further improve the molecular weight, a prolonged reaction time is necessary. Nonetheless, these results suggest the BHET obtained *via* glycolysis of PET can be used as monomer for PET production. To reinforce this claim, in‐depth polymerization studies with BHET are necessary.

**TABLE 5:** Results for the methanolysis and glycolysis using TMG_2_e of post‐consumer waste samples at 180°C.

Waste sample	*m* (g)	*n*_PET_ (mmol)	*c*_cat_ a (mol%)	Nucleophile	*t* (h)	*Y* b (%)
Mixed plastic waste Spain^c^	1.0	0	‐d	EG	72	0
Mixed plastic waste (VFB)	2.25	3.5	2.0	MeOH	5	54
Mixed palstic waste (VFB)^c^	1.0	1.5	2.6	EG	72	9
PET bottles^c^	1.0	5.2	0.8	EG	72	11
PET bottles	0.67	3.5	2.0	EG	24	26
PET trays	0.67	3.0	2.3	MeOH	5	52
PET trays^c^	1.0	4.5	0.9	EG	72	22
PET trays	0.67	3.0	2.3	EG	24	39
PET trays	3.0	16	2.3	EG	51	55
PET trays [84]	7.0	32	1.1	EG	74	49

a Regarding the PET repeating units.

b Isolated yield.

c The reaction was stirred at 140°C for 18 h prior. After no depolymerization was observed, the temperature was increased to 180°C.

d 1 wt% regarding the waste sample of catalyst was added.

### 2.8 Recycling Mixed Plastics

The separation of plastic mixes, especially waste material, is one of the major challenges for a circular plastics economy. Sequential recycling is a promising approach to achieve high‐quality products from mixed plastics [3, 5, 46, 63, 85, 86]. A recent study using an imino‐pyrrole Zn complex for the sequential recycling of binary mixtures of PLA, PET, and bisphenol A polycarbonate showed promising results for efficient polymer separation [46]. Being able to selectively depolymerize one polymer depending on the temperature enables enhanced treatment of plastic waste streams in a cascade recycling approach [63]. Removing PLA from a PET waste stream could lead to improved mechanical recycling of PET [19, 63, 65, 87]. We investigated the cascade recycling of mixed plastics by creating different polymer mixes with equimolar amounts of each polymer (Figure S17): PLA and PET; PLA and PCL; PCL and PET; and PLA, PET, and PCL.

**FIGURE 5:**
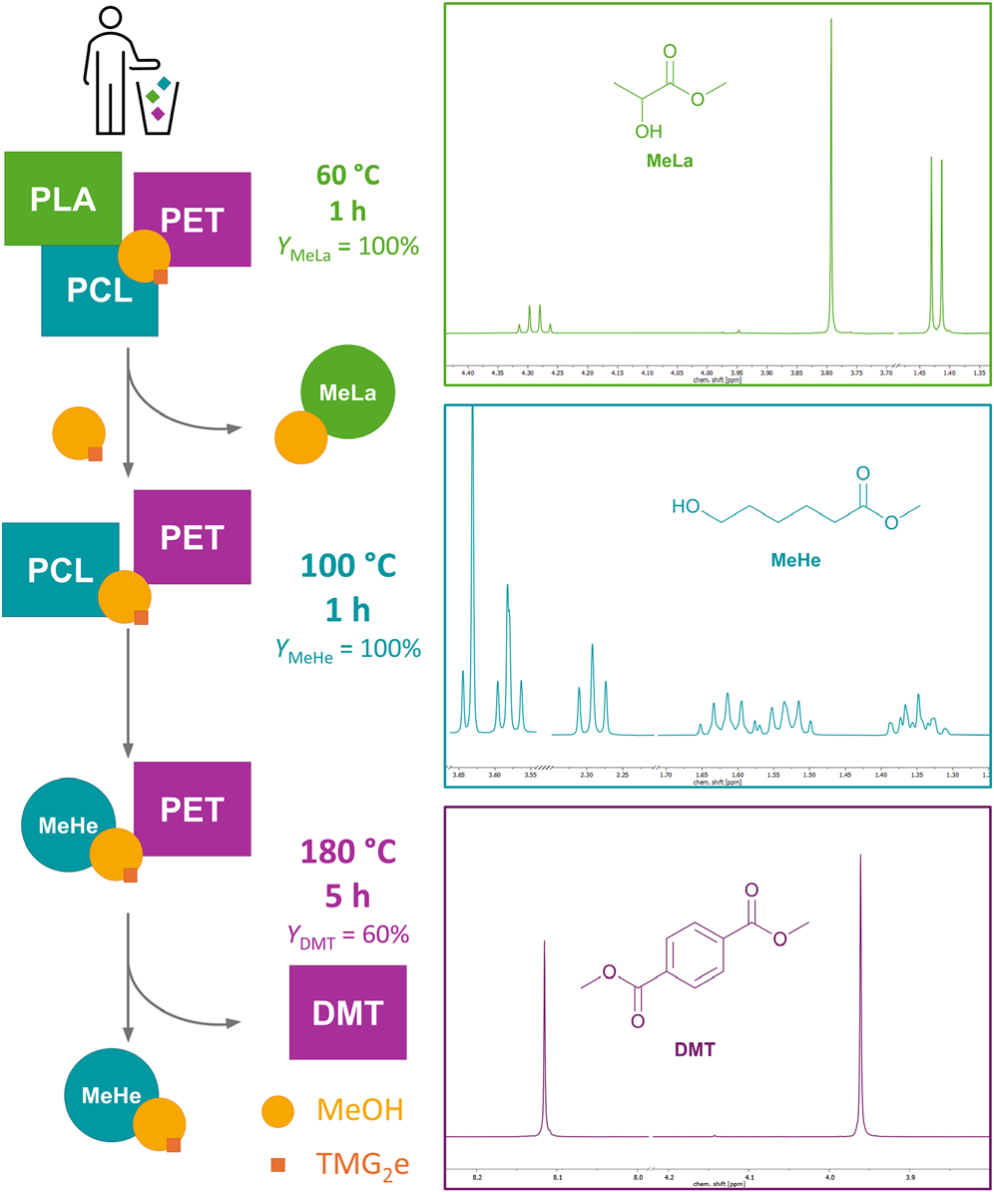
Temperature‐controlled cascade recycling of a PLA, PCL, and PET mix at 60, 100, and 180°C.

The sequential degradation with and without removal of the volatile components was evaluated at different temperatures (*T*_PLA_ = 60°C, *T*_PCL_ = 100°C, *T*_PET_ = 180°C) as well as a simultaneous approach at 180°C. Further, we investigated the influence of the physical form of PLA using PLA as powder (Ø = 0.75 mm) or film (0.5 × 0.5 cm). PCL was used as pellets (Ø = 3 mm) and PET as powder (Ø = 0.75 mm). In Table 6 and Table S7, an overview of the results of the experiments performed is provided. In contrast to PLA and PCL, PET is not dissolved during the reaction but rather depolymerized to soluble oligomers and DMT. Thus, DMT was isolated to determine the *Y*_dp_. For the other two polymers, ^1^H NMR spectroscopic analysis was deployed to determine the corresponding *Y*_dp_ because the small scale aggravates the isolation process. The isolation of MeLa from MeOH was performed successfully for the large‐scale experiments described in Table 3 and for MeHe a successful isolation process was reported by Cheung et al. [22]. The performed experiments in Table 6 show that TMG_2_e can easily depolymerize PLA and PET in the presence of other polymers at different temperatures. However, the methanolysis of PCL seems to be hindered in the presence of PLA, MeLa, PET, and/or DMT. Furthermore, the methanolysis of ternary polymer mixes was strongly influenced by the physical form of PLA. An in‐depth evaluation of this study is provided in Section S8.1 in the Supporting Information. To improve the PCL conversion, first, we depolymerized PLA from the PLA, PCL, and PET mix at 60°C. After 95 min, complete conversion of PLA to MeLa (*Y*_MeLa_ = 100%) was observed and all volatile components (MeLa, MeOH) were removed. To start the depolymerization of PCL, MeOH (4 mL) was added and the reaction tube was placed in an oil bath at 100°C. After 2 h at 100°C, we could only detect a *Y*_MeHe_ of 12%. Thus, we increased the temperature to 180°C to start the depolymerization process of PET. After 5 h, the reaction mixture was turbid and yellow. ^1^H NMR spectroscopic analysis showed slow depolymerization of PCL (*Y*_MeHe_ = 19%) as well as depolymerization of PET to DMT. PET particles were still clearly visible in the reaction mixture. Thus, we prolonged the reaction time to 22.5 h, after which a clear solution was observed. Resonances for PCL were still observable in the ^1^H NMR spectrum (*Y*_MeHe_ = 41%), but PET was depolymerized completely to DMT and oligomeric species. The oligomeric species were not further identified and are likely the cause of the discoloration as observed during the glycolysis of PET. The isolation process of DMT was performed by adding deionized water to the obtained off‐white solid after cooling the reaction down to room temperature. The mixture was filtered, washed with deionized water, and dried in high vacuum. The obtained colorless DMT was contaminated with PCL, MeHe, and oligomeric species of PET. These results suggest that the catalyst is deactivated and/or removed during the isolation process of MeLa and MeOH. A possible way to prevent the severe activity loss could be the immobilization of the catalyst [88]. Based on the results obtained, we developed the temperature‐controlled cascade recycling approach displayed in Figure 5.

**TABLE 6:** Results for the methanolysis (4 mL MeOH) of plastic mixes using 2 mol% TMG_2_e regarding PLA or PCL ester bonds, respectively 1 mol% TMG_2_e regarding PET ester bonds.

Polymers^a^	*T* (°C)	*t* b (h)	*Y*_MeLa_ c (%)	*Y*_MeHe_ d (%)	*Y*_DMT_ e (%)
PLA, PCL	60	1	100	24	–
	100	1	100	74	–
	100	20	100	79	–
PLA, PET	60	1	100	–	–
	180	5	100	–	73
PCL, PET	100	1	–	86	–
	180	5	–	95	80
PLA, PCL, PET	180	4.5	100	18	n.d.^f^
PLA, PCL, PET^g^	95	1	100	^−^	–
	100	2	–	12	–
	180	22.5	–	41	n.d.^f^
PLA, PCL, PET^g^ (Figure 5)	60	1	100	–	–
	100	1	–	100	–
	180	5	–	100	60

a PLA (250 mg, 3.5 mmol), PCL (3.4–3.5 mmol), and PET (3.5 mmol) were applied according to the stated content of the polymer mix.

b Time at given temperature.

c Determined with ^1^H NMR spectroscopic analysis and calculated according to literature [28].

d Determined with ^1^H NMR spectroscopic analysis and calculated according to literature [22].

e DMT was isolated from a clear, homogeneous reaction mixture indicating complete depolymerization of PET to side products or DMT.

f DMT could not be isolated without traces of other depolymerization products, but a clear reaction mixture was obtained after the given reaction time indicating complete depolymerization of PET.

g Removal of MeLa and MeOH after complete depolymerization of PLA at 60°C.

Here, the addition of TMG_2_e after the removal of MeLa and MeOH compensates the previously described activity loss: After 1 h, complete depolymerization of PLA was observed. MeLa and MeOH were removed in high vacuum, MeOH (4 mL) and TMG_2_e (2 mol% regarding the PCL ester bonds) were added to the remaining reaction mixture, and the reaction was started at 100°C. After 1 h, PCL was depolymerized completely to MeHe (*Y*_MeHe_ = 100%). The high conversion of PCL in the presence of PET, might be due to the increased catalyst concentration in the reaction mixture. After 5 h at 180°C, DMT could be isolated in high purity from MeHe and MeOH in a good yield (*Y*_DMT _= 60%) following the previously described isolation process. The procedure displayed in Figure 5 allows selective, temperature‐controlled alcoholysis of specific polymers from plastic mixes. The separation of PLA from PET and other polymers could facilitate effective mixed waste stream treatment and recycling. For example, after the chemical recycling of PLA at mild reaction conditions, PET can be easily separated and reintroduced into the mechanical recycling cycle [63]. Further improvements on the isolation of MeLa and MeOH after the depolymerization of PLA or the immobilization of the catalyst could prevent the second addition of catalyst.

### 2.9 Post‐Consumer Plastic Waste Depolymerization

To further confirm the applicability of TMG_2_e in industry, post‐consumer plastic waste was investigated. For PLA, we tested the methanolysis of single‐use poly‐L‐lactide (PLLA) cups (Figure S19) [21]. The PLLA cups contained water, cola, and cold coffee. After use, the PLLA cups were not cleaned and cut into small pieces (0.5 × 0.5 cm). Using 0.5 mol% TMG_2_e, we were able to depolymerize PLA to MeLa within 60 min (*Y*_MeLa_ = 98%) under standard reaction conditions (3.47 mmol PLA, 0.5 mol% TMG_2_e, 7.1 equiv MeOH, 4 mL THF, 60°C, 260 rpm). TMG_2_e showed no significant activity loss due to the remains of the beverages. To obtain a more realistic insight into post‐consumer waste depolymerization, we investigated the glycolysis and methanolysis of PET from waste material collected in Spain and Denmark (Figure S20). Four different post‐consumer waste sources with varying amounts of PET were tested (Table 5): Mixed plastic waste from Spain containing no PET (AIMPLAS), mixed plastic waste from Denmark (VFB, 30% PET, 41% polyethylene, 3% polystyrene, 9% polypropylene, 8% multilayer brick, 9% nonplastic material), PET trays (AIMPLAS, 87% PET, 11% PE), and PET bottles (AIMPLAS, 100% PET). Using MeOH, TMG_2_e depolymerized the waste material from PET trays to DMT (*Y*_DMT_ = 52%) and from mixed plastic waste (VFB, *Y*_DMT_ = 54%) within 5 h at 180°C. We obtained a lower amount of DMT compared to pristine PET (*Y*_DMT_ = 73%) and discoloration of the product was observable. Nonetheless, ^1^H NMR spectroscopic analysis suggests we obtained DMT in high purity. During the isolation of DMT from the remaining plastic particles for the mixed plastic waste material (VFB), we were able to obtain DMT as crystalline colorless powder by performing additional purification steps, which decreased the amount of obtained DMT significantly. Thus, more advanced technical purification processes as done in industry need to be applied to enhance the color and yield of the obtained DMT. Besides discoloration of the product, the waste material leads to a discoloration of the reaction mixture; thus, we could not observe a clear solution indicating complete PET consumption. Therefore, we choose elongated reaction times for the glycolysis compared to pristine PET material to ensure the complete depolymerization of PET.

The results listed in Table 5 show that waste materials could influence the activity of TMG_2_e. The mixed plastic waste from VFB shows a lower yield of BHET than PET waste from plastic bottles or plastic trays. The low PET content and the high content of other materials and impurities aggravate the isolation process further, as described for the isolation of DMT. Another factor impacting the BHET yields is the crystallinity of the PET. PET bottles have a higher crystallinity than the PET used in the trays, which could lead to lower conversion of PET. We could further increase the BHET yield by increasing the catalyst loading to 1.0 or 1.3 mol% regarding the PET ester bonds. Hot filtration of the reaction mixture improved the BHET yield by up to 55%. The glycolysis of the PET trays showed the highest BHET yield under the given reaction conditions. We successfully scaled up the PET glycolysis of the PET trays reaching *Y*_BHET_ = 55% from 3 g of post‐consumer PET waste and 49% BHET from 7.0 g of post‐consumer PET waste. The produced recycled BHET was successfully used as a monomer for PET production as described previously [84]. We demonstrated that TMG_2_e is a versatile and robust catalyst applicable in the chemical recycling of post‐consumer PET waste and other post‐consumer polyester waste: closing the loop of PET production, consumption, and recycling to monomers for new PET production.

## 3 Conclusions

In this study, we investigated three robust bisguanidine organocatalysts for their applicability in a sustainable, circular plastics economy. Evaluation of the structure–reactivity relationship between the length of the linker between two guanidine functionalities and the activity of the catalysts in the methanolysis and ethanolysis of PLA established the ethylene‐bridged TMG_2_e as the most active guanidine organocatalyst depolymerizing PLA within 3 min under mild reaction conditions (*k*_dp_ = 10.3·10^−1 ^mol%^−1^ min^−1^). At elevated temperatures (150°C) and under solvent‐free conditions, we achieved a complete methanolysis of PLA to MeLa (*Y*_MeLa_ = 98%) within 5 min. Thus, we studied TMG_2_e under industrially relevant conditions by applying the principles of Green Chemistry. The scalability of the methanolysis and ethanolysis was demonstrated as well as the recyclability of the nucleophiles used. Besides PLA, TMG_2_e depolymerized other polyesters (PCL, PET) efficiently. Further, TMG_2_e was successfully applied in the depolymerization of not only pristine polymers and challenging polymer mixes (PLA, PCL, PET) but also in the depolymerization of post‐consumer plastic waste, thus proving its high robustness and activity. However, we see a decrease in the activity using post‐consumer waste. The influence of the varying composition of the waste material needs to be addressed and further studied to identify and possibly circumvent the loss in catalytic activity. Overall, the herein presented straightforward and robust process to transform even hard‐to‐recycle mixed materials into a source for new, value‐added chemicals or into a new feedstock for monomers makes TMG_2_e a key enabler for a circular plastics economy.

## 4 Experimental

### 4.1 Resynthesis of Bis(*N*,*N*,*N′*,*N′*‐tetramethylguanidino)ethane (TMG_2_e)

TMG_2_e was synthesized accordingto literature [67]. The synthesis was performed with synthesis‐grade reagents and without a protective atmosphere. A solution of tetramethylchloroformamidinium chloride Vilsmeier salt (11.0 g, 64.3 mmol, 2.17 equiv) in MeCN (80 mL) was added dropwise to a solution of ethane‐1,2‐diamine (1.80 g, 2.00 mL, 29.7 mmol, 1.00 equiv) and NEt_3_ (8.80 mL, 63.1 mmol, 2.13 equiv) in MeCN (100 mL) at 0°C. The mixture was treated to reflux conditions for 3 h at 110°C. After cooling to room temperature, a solution of sodium hydroxide (2.50 g, 62.5 mmol, 2.11 equiv) in water (30 mL) was added and the solvent was removed under reduced pressure. The residue was transferred to a separating funnel using MeCN (75 mL) and deionized water (55 mL). A solution of potassium hydroxide (30.3 g, 540 mmol, 18.2 equiv) in deionized water (30 mL) was added. The phases were separated, and the aqueous phase was extracted with MeCN (3 × 50 mL). Remaining water in the combined organic phases was removed over Na_2_SO_4_ and filtered over activated charcoal and celite. The solvent was removed under reduced pressure. Tetramethyl urea was removed under high vacuum at 60°C. The product was further purified by sublimation (stationary vacuum at 4·10^−2^ mbar, 135°C). TMG_2_e was obtained as a colorless crystalline solid (*Y* = 71%, *Y*_Lit._= 72% [67]). The obtained FAIR data is provided under: doi: 10.14272/reaction/SA‐FUHFF‐UHFFFADPSC‐ZCBQJRXSMA‐UHFFFADPSC‐NUHFF‐NUHFF‐NUHFF‐ZZZ.

^**1**^ **H NMR** (400 MHz, CDCl_3_): 3.30 (s, 4H), 2.76 (s, 12H), 2.67 (s, 12H) ppm.

### 4.2 Synthesis of 1,2‐Di[*2N*‐(1,1,3,3‐tetramethylguanidinium)]ethane Dichloride ([TMG_2_eH_2_]^2+^ 2Cl^−^)

TMG_2_e (273 mg, 1.07 mmol, 1.00 equiv) was dissolved in MeCN (2.00 mL). Concentrated hydrochloric acid (1.05 g, 880 μL, 10.6 mmol, 9.98 equiv) was added and the reaction stirred at room temperature for 1.5 h. All volatile components were removed under high vacuum at 60°C. The obtained colorless solid was washed with MeCN and the remaining solvent was removed under reduced pressure. The hydrochloride of TMG_2_e was obtained as colorless powder (169 mg, 421 μmol, 40% yield). The obtained FAIR data is provided under: doi: 10.14272/reaction/SA‐FUHFF‐UHFFFADPSC‐LYPYBITZWV‐UHFFFADPSC‐NUHFF‐NUHFF‐NUHFF‐ZZZ.

^**1**^ **H NMR** (400 MHz, MeCN‐*d*_3_): δ = 9.17 (s, 2H), 3.52–3.44 (m, 4H), 2.99 (b, 24H) ppm.

^**1**^ **H NMR** (400 MHz, DMSO‐*d*_6_): δ = 8.10 (s, 2H), 3.39–3.36 (m, 4H), 2.91 (b, 24H) ppm.

^**1**^ **H NMR** (400 MHz, CDCl_3_): δ = = 8.10 (s, 2H), 3.39–3.36 (m, 4H), 2.91 (b, 24H) ppm.

^**13**^ **C NMR** (400 MHz, CDCl_3_): δ = 162.00 (CN_3_), 44.49 (CH_2_), 40.65 (CH_3_) ppm.

**ESI MS:** *m/z*: [M‐H]^+^: Calculated for [C_12_H_29_N_6_]^+^: 257.24482 g mol^−^^1^. Found for [C_12_H_29_N_6_]^+^: 257.24442 g mol^−^^1^.

### 4.3 Synthesis of Bis(*N*,*N*,*N′*,*N′*‐tetramethylguanidino)pentane (TMG_2_pe)

TMG_2_pe was synthesized according to literature [67, 68, 69]. Tetramethylchloroformamidinium chloride Vilsmeier salt (5.40 g, 31.5 mmol, 2.07 equiv) was dissolved in absolute MeCN (60 mL). The solution was added dropwise to a solution of pentane‐1,5‐diamine (1.56 g, 15.2 mmol, 1.00 equiv) and NEt_3_ (3.19 g, 4.39 mL, 31.5 mmol, 2.07 equiv) in absolute MeCN (30 mL) at *T* = 0°C. The mixture was heated to reflux conditions (*T*_oil bath_ = 110°C) for 3 h. After cooling down, a solution of NaOH (1.26 g, 31.5 mmol, 2.07 equiv) in water (15 mL) was added. The solvent was removed under reduced pressure. The residue was transferred to a separating funnel using MeCN (40 mL). A solution of KOH (15.0 g, 267 mmol, 17.6 equiv) in water (15 mL) was added. The different layers were separated, and the aqueous layer was extracted twice with MeCN (2 × 40 mL). Remaining water in the organic layer was removed over NaSO_4_ and filtered over activated charcoal. The solvent was removed under reduced pressure and tetramethyl urea was removed under high vacuum at 60°C. The crude product was obtained as partly liquid and partly solid slightly yellow product phases. For further purification, recrystallization with *n*‐hexane was conducted. The product mixture was stored in the fridge for 10 days. Subsequently, the liquid and solid phases were separated. The solid was washed with *n*‐pentane several times. TMG_2_pe was obtained as colorless oil (*Y* = 40%). The obtained FAIR data is provided under: doi: https://dx.doi.org/10.14272/reaction/SA-FUHFF-UHFFFADPSC-VKARKQYNUZ-UHFFFADPSC-NUHFF-NUHFF-NUHFF-ZZZ.

^**1**^ **H NMR** (400 MHz, CDCl_3_): δ = 3.08 (t, *J* = 7.0 Hz, 4H, CH_2_), 2.71 (s, 12H, CH_3_), 2.68 (s, 12H, CH_3_), 1.51–1.47 (m, 4H, CH_2_), 1.39–1.26 (m, 2H, CH_2_) ppm.

^**13**^ **C NMR** (400 MHz, CDCl_3_): δ = 159.96 (CN_3_), 49.84 (CH_2_N), 39.76 (CH_3_), 38.97 (CH_3_), 32.88 (CH_2_CH_2_N), 25.60 (CH_2_CH_2_CH_2_N) ppm.

**ESI‐MS** *m/z*: [M+H]^+^: Calculated for [C_15_H_35_N_6_]^+^: 299.29177 g mol^–1^. Found for [C_15_H_35_N_6_]^+^: 299.29118 g mol^–1^.

**FTIR** (ATR, ṽ) = 2999 (vw), 2922 (w), 2901 (w), 2859 (w), 2839 (w), 2797 (w), 1616 (vs), 1493 (w), 1449 (w), 1437 (w), 1425 (w), 1402 (vw), 1358 (vs), 1308 (vw), 1233 (w), 1132 (m), 1103 (vw), 1063 (w), 1055 (w), 991 (w), 912 (w), 746 (vw), 579 (vw) cm^–1^.

### 4.4 PLA Alcoholysis

#### 4.4.1 General Procedure

Catalyst (1.0 mol%) and THF (4 mL) were provided in a Young‐type Schlenk tube. PLA (250 mg, 3.47 mmol, 1.00 equiv) was added. The reaction mixture was dissolved using an external heat source, placed in a heated oil bath (*T* = 60°C), and stirred at 260 rpm. The reaction was started (*t* = 0 min) by addition of MeOH (1.0 mL, 7.1 equiv) or EtOH (1.4 mL, 6.9 equiv). The reaction process was monitored using ^1^H NMR spectroscopy.

#### 4.4.2 Determination of *k*_dp_, *E*_A_, Δ*S* ^≠^, and Δ*H* ^≠^

The alcoholysis was performed under inert conditions using Schlenk techniques. The catalyst (0.1–2.0 mol%) and THF (4 mL) were provided in a Young‐type Schlenk tube. PLA (film (0.5 × 0.5 cm), 250 mg, 3.47 mmol, 1.00 equiv) was added in an Ar counterflow. The reaction mixture was dissolved using an external heat source, placed in a heated oil bath (*T* = 30–60°C), and stirred at 260 rpm. The reaction was started by addition of the nucleophile (*t *= 0 min): MeOH (1.0 mL, 7.1 equiv) or EtOH (1.4 mL, 6.9 equiv). The reaction process was monitored using ^1^H NMR spectroscopy. Samples were taken in appropriate time intervals.

#### 4.4.3 Solvent‐Free Methanolysis

PLA (250 mg, 3.47 mmol, 1.00 equiv), TMG_2_e (8.9 mg, 35 μmol, 0.010 equiv), and MeOH (1.00 mL, 24.7 mmol, 7.13 equiv) were placed in a preheated oil bath at 150°C (*t* = 0 min) and stirred at 260 rpm. The reaction process was monitored using ^1^H NMR spectroscopy. After 5 min, *Y*_MeLa_ = 98% was determined using ^1^H NMR spectroscopic analysis. Additional information on the reaction procedure and original analysis data files are available *via* the Chemotion repository: 10.14272/reaction/SA‐FUHFF‐UHFFFADPSC‐LPEKGGXMPW‐UHFFFADPSC‐NUHFF‐NUHFF‐NUHFF‐ZZZ.8.

#### 4.4.4 Scale‐up of PLA Alcoholysis

PLA film (0.5 × 0.5 cm) and TMG_2_e (0.5–1.0 mol%) were provided in a Schlenk flask, excess alcohol was added and the reaction heated to 60°C for the methanolysis or 60–100°C for the ethanolysis. The reaction was stopped after all polymer particles were dissolved. All volatile components were removed under high vacuum and collected in a cooling trap. The alcohol was removed from the obtained clear solution using rotary evaporation. The alkyl lactates were obtained as clear, colorless liquids.

### 4.5 PCL Methanolysis

PCL (pellets (Ø = 3 mm), 396 mg, 3.47 mmol, 1.00 equiv) and TMG_2_e (17.8 mg, 69.4 µmol, 1.99 mol%) were provided in a Young‐type Schlenk tube. MeOH (4 mL) was added and the tube placed in a preheated oil bath at 100°C (*t* = 0 min). The reaction process was monitored using ^1^H NMR spectroscopy. The tube was cooled to room temperature to take samples in appropriate time intervals. Afterward, the tube was placed in the oil bath again.

### 4.6 PET Methanolysis

#### 4.6.1 Pristine PET

PET (powder (Ø = 0.75 mm), 667 mg, 3.47 mmol, 1.00 equiv) and TMG_2_e (17.8 mg, 69.4 µmol, 2.00 mol%) were provided in a Young‐type Schlenk tube. MeOH (4 mL) was added and the tube placed in a preheated oil bath at 180°C (*t* = 0 min). The tube was cooled to room temperature to take samples in appropriate time intervals. Afterward, the tube was placed in the oil bath again. After a clear solution was obtained, the reaction was cooled to room temperature upon which a colorless solid formed; deionized water (10 mL) was added. The mixture was stored at 4°C. The colorless solid was filtered and washed with deionized water. Dimethyl terephthalate was obtained as colorless solid.

#### 4.6.2 Post‐Consumer PET Waste

Post‐consumer waste material (0.67 g) and TMG_2_e (2.3 mol%) were provided in a Young‐type Schlenk tube. MeOH (4.0 mL) was added, and the tube was placed in a preheated oil bath at 180°C (*t* = 0 min). After 5 h, the reaction was cooled to room temperature, and deionized water (10 mL) was added. The mixture was filtered and the resulting solid was dried under high vacuum. The obtained dimethyl terephthalate was obtained as gray to colorless solid.

### 4.7 PET Glycolysis

#### 4.7.1 Pristine PET

PET (powder (Ø = 0.75 mm), 667 mg, 3.47 mmol, 1.00 equiv) and TMG_2_e (17.8 mg, 69.4 µmol, 2.00 mol%) were provided in a Young‐type Schlenk tube. Ethylene glycol (4 mL) was added, and the tube was placed in a preheated oil bath at 180°C (*t* = 0 min). After the desired reaction time, the reaction was cooled to room temperature, and deionized water (10 mL) was added. The mixture was rapidly filtered, and the filtrate was stored at 4°C. Bis(2‐hydroxyethyl) terephthalate was obtained in moderate yield as light gray solid.

#### 4.7.2 Post‐Consumer PET Waste

Post‐consumer waste material and TMG_2_e were provided in a Young‐type Schlenk tube. Ethylene glycol (2.0 mL) was added, and the tube was placed in a preheated oil bath at 180°C (*t* = 0 min). After the desired reaction time, the reaction was cooled to room temperature, and deionized water (10 mL) was added. The mixture was filtered, and the filtrate was stored at 4°C. Bis(2‐Hydroxyethyl) terephthalate was obtained in low to good yield as gray solid.

#### 4.7.3 Scale‐up of PET Glycolysis

Pristine PET (powder (Ø = 0.75 mm)) or post‐consumer waste PET and TMG_2_e (1–2 mol%) were provided in a Schlenk flask. Ethylene glycol was added, and the flask was placed in a preheated oil bath at 180°C (*t* = 0 min). After a certain reaction time, the reaction was cooled down and deionized water was added. The mixture was rapidly filtered, and the filtrate was stored at 4°C. Bis(2‐hydroxyethyl) precipitated as brown solid. It was filtered and dried in high vacuum. Bis(2‐hydroxyethyl) was obtained in a good yield as gray solid.

### 4.8 Methanolysis of Polymer Mixes

#### 4.8.1 Without Removal of Volatile Components

The mixes were prepared from PLA (film (0.5 × 0.5 cm), 3.5 mmol, 1.0 equiv) or PLA (powder (Ø = 0.75 mm), 3.5 mmol, 1.0 equiv), PCL (pellets (Ø = 3 mm), 3.5 mmol, 1.0 equiv), and PET (powder (Ø = 75 mm), 3.5 mmol, 1.0 equiv) accordingly. TMG_2_e (17.8 mg, 69.4 μmol, 1.98 mol% of PLA ester bonds) and MeOH (4 mL) were added. The reaction was heated to 60°C (*t*_1 _= 0 min), 100°C (*t*_2_ = 0 min), and/or 180°C (*t*_3_  = 0 min) for appropriate time intervals. The reaction was monitored using ^1^H NMR spectroscopy. Methyl lactate and methyl hexanoate were not further isolated. If PET was used, the reaction was cooled to room temperature, and the formation of a colorless solid was observed. The mixture was filtered. The obtained colorless solid was washed with MeOH or deionized water. Dimethyl terephthalate was dried in high vacuum and obtained as colorless solid.

#### 4.8.2 With Removal of Volatile Components

The mixes were prepared from PLA (film (0.5 × 0.5 cm), 3.5 mmol, 1.0 equiv), PCL (pellets (Ø = 3 mm), 3.5 mmol, 1.0 equiv), and PET (powder (Ø = 75 mm), 3.5 mmol, 1.0 equiv). TMG_2_e (17.8 mg, 69.4 μmol, 1.98 mol% of PLA ester bonds) and MeOH (4 mL) were added. The reaction was heated to 60°C (*t*_1 _= 0 min). After depolymerization of PLA, all volatile components were removed in high vacuum. Methyl lactate and MeOH were collected in a cooling trap. TMG_2_e (17.8 mg, 69.4 μmol, 1.98 mol%) and MeOH (4 mL) were added to the reaction flask which was placed in an oil bath at 100°C (*t*_2 _= 0 min), and/or 180°C (*t*_3 _= 0 min) for appropriate time intervals. The reaction was monitored using ^1^H NMR spectroscopy. After a homogeneous reaction mixture was observed, the reaction was cooled to room temperature, and the formation of a colorless solid was observed. The mixture was filtered. The obtained colorless solid was washed with MeOH or deionized water. Dimethyl terephthalate was dried in high vacuum and obtained as colorless solid. Methyl hexanoate in the filtrate was not further isolated.

## Supporting Information

Additional supporting information can be found online in the Supporting Information section. The authors have cited additional references within the Supporting Information.

## Funding

This work was supported by European Commission (953073), Deutsche Forschungsgemeinschaft (ID: 390919832) and Fuel Science Cluster.

## Conflicts of Interest

The authors declare no conflicts of interest.

## Supporting information

Supplementary Material

## Data Availability

Original NMR data for the kinetic experiments performed are available *via* the RADAR4Chem repository by FIZ Karlsruhe – Leibniz Institut für Informationsinfrastruktur and are published under an Open Access model (CC BY‐NC‐SA 4.0 Attribution‐NonCommercial‐Share Alike: https://www.radar-service.eu/radar/en/dataset/2ejvm338fzgnukrr. The data for all other experiments can be found in the Chemotion Repository: https://dx.doi.org/10.14272/collection/LB_2024‐12‐11.
