# Supplementary material for: Open‐ and Closed‐Loop Recycling of Polyesters and Post‐Consumer Waste Under Industrially Relevant Conditions Using Bisguanidine Organocatalysts
Source: ChemSusChem. 2026 Jan 12;19(1):e202502062. doi: 10.1002/cssc.202502062 (PMC12796559; doi:10.1002/cssc.202502062)
Supplement: Supplementary file 1 — Supplementary Material [file CSSC-19-e202502062-s001.pdf]

## Supplementary information

# Open- and closed-loop recycling of polyesters and post-consumer waste under industrially relevant conditions using bisguanidine organocatalysts

Lisa Burkart, Alisa Hahn, Yasemin Kara, Damon Blum, Alexander Hoffmann and Sonja Herres-Pawlis

Institute of Inorganic Chemistry, RWTH Aachen University, Landoltweg 1a, 52074 Aachen (Germany).

## Table of contents


1. Analytical methods 4

1.1. Nuclear magnetic resonance (NMR) spectroscopy 4

1.2. Electrospray Ionization Mass Spectrometry (ESI-MS) 4

1.3. Fourier-Transform Infrared Spectroscopy (FTIR) 4

1.4. Differential Scanning Calorimetry (DSC) 4

1.5. General synthesis information 4

2. Evaluation of the depolymerizations of polyesters 5

2.1. The alcoholysis of PLA 5

2.2. Evaluation of the methanolysis of PLA 7

2.3. $TMG_2e$ and $TMG_2p$ vs. [$FeCl_2(TMG_2e)$] and [$FeCl_2(TMG_2p)$] 7

2.4. Results for the investigation of the influence of the linker length 8

2.5. Methanolysis using $TMG_2eH_2Cl_2$ 8

2.6. Methanolysis using $TMG_2e$ and $TMG_2eH_2Cl_2$ (1:1) 8

2.7. Evaluation of the ethanolysis of PLA 9

2.8. Evaluation of the methanolysis of PCL 9

2.9. Methanolysis of PCL in presence of MeLa 10

2.10. Evaluation of the methanolysis of PET 10

2.11. Evaluation of the glycolysis of PET 10

2.12. Control reaction using EG and $TMG_2e$ 10

2.13. Control reactions using BHET and THF 10

2.14. Control reactions using BHET, $TMG_2e$, and THF 10

2.15. Control reactions using BHET, $TMG_2e$, and EG 10

3. Determination of $k_{dp}$ for the methanolysis and ethanolysis of PLA 11

3.1. Determination of $k_{dp}$ for the methanolysis of PLA using $TMG_2e$ as catalyst 11

3.2. Determination of $k_{dp}$ for the methanolysis of PLA using $TMG_2p$ as catalyst 11

3.3. Determination of $k_{dp}$ for the methanolysis of PLA using TMG2pe as catalyst 12

3.4. Determination of $k_{dp}$ for the ethanolysis of PLA using $TMG_2e$ as catalyst 12

3.5. Determination of $k_{dp}$ for the ethanolysis of PLA using $TMG_2p$ as catalyst 13

4. Determination of $E_A$, $\Delta H^{\neq}$, and $\Delta S^{\neq}$ for the methanolysis and ethanolysis of PLA 13

4.1. Methanolysis of PLA at different $T$ using $TMG_2e$ as catalyst 14

4.2. Methanolysis of PLA at different $T$ using $TMG_2p$ as catalyst 15

4.3. Methanolysis of PLA at different $T$ using $TMG_2pe$ as catalyst 16

4.4. Ethanolysis of PLA at different $T$ using $TMG_2e$ as catalyst 17
4.5. Ethanolysis of PLA at different $T$ using $TMG_2p$ as catalyst 18
5. Determination of $k_{app}$ of the methanolysis using TMG 18
6. Control experiments and solubility behavior 19
6.1. Methanolysis under standard reaction conditions without a catalyst 19
6.2. Control experiment using no catalyst and investigation of the solubility behavior of PLA in EtOH 19
7. Large-scale methanolysis and ethanolysis of PLA 19
8. Depolymerization of mixed plastics 20
8.1. In-depth evaluation of the performed (cascade) recycling experiments 21
9. Depolymerization of commercially available PLLA cups 22
10. Post-consumer waste 22
10.1. Composition of post-consumer waste 22
10.2. Depolymerization of post-consumer waste 23
11. Characteristics of used polymers 24
11.1. Differential scanning calorimetry 24
11.2. PLA film 24
11.3. PLA powder 24
11.4. PET powder 25
11.5. Infrared spectroscopy 25
11.6. PLA film 25
11.7. PLA powder 26
11.8. PLA cup 26
11.9. PCL pellets 27
12. References 27

# 1. Analytical methods

## 1.1. Nuclear magnetic resonance (NMR) spectroscopy

Nuclear magnetic resonance (NMR) spectra were recorded on a Bruker Avance Neo 400 or Bruker Avance III HD 400 nuclear resonance spectrometer. The resonances were referenced to the residual proton signals of the deuterated solvent ($\delta_H$($CDCl_3$) = 7.26 ppm), ($\delta_H$(MeCN-$d_3$) = 1.94 ppm) and ($\delta_H$(DMSO-$d_6$) = 2.50 ppm) relative to TMS.[1] Data in the $^1$H and $^{13}$C NMR spectra are stated as follows: chemical shift (δ in ppm) (multiplicity, coupling constant (J in Hz), integration). To describe the multiplicity of the signals the following abbreviations are used: s = singlet, d = doublet, t = triplet, q = quartet, m = multiplet or combinations thereof. For the Bruker Avance III HD 400 the software Topspin (Version 3.5.7) from Bruker and for the Bruker Avance Neo 400 the software TopSpin (Version 4.2.0, 4.4.1) from Bruker were used for data acquisition. For visualization and examination of the NMR spectra the software MestReNova (Version 12.0.1-20560) from Mestrelab Research was used.[2] The $^1$H NMR spectra were processed using auto phase correction and a baseline correction (Whittaker Smoother). If necessary, adjustments were performed using the manual phase correction.

## 1.2. Electrospray Ionization Mass Spectrometry (ESI-MS)

ESI-MS of the iron complexes was performed on an UHR-TOF Bruker Daltonik maXis II, an ESI-quadrupole time-of-flight (qToF) mass spectrometer capable of a resolution of at least 80.000 FWHM and a source voltage of 4.5 kV. Detection was in positive ion mode and acetonitrile was used as solvent. The mass spectrometer was calibrated subsequently to every experiment *via* direct infusion of a L-proline sodium salt solution, which provided a *m/z* range of singly charged peaks up to 3000 Da in both ion modes. ESI-MS was performed to further confirm the successful synthesis of $TMG_2$pe and $[TMG_2eH_2]^{2+}$ $2Cl^-$.

## 1.3. Fourier-Transform Infrared Spectroscopy (FTIR)

FTIR spectra were recorded with a Shimadzu IRTracer-100 using a CsI beam splitter with an ATR unit (Quest model from Specac utilizing a robust monolithic crystalline diamond) with a resolution of 2 $cm^{-1}$ (number of scans = 40, range = 200–4000 $cm^{-1}$). For data collection and the baseline correction, the software LabSolutions IR[3] was used. The peak picking and the assignment of intensities was carried out with the Spectra Editor integrated in the electronic lab notebook Chemotion.[4]

## 1.4. Differential Scanning Calorimetry (DSC)

DSC curves of selected polymer samples were recorded on a Netzsch DSC 204 F1 Phoenix equipped with an intra-cooler. The samples were weighed into 50 µL aluminum pans and sealed with punctuated aluminum lids. For all measurements, three cycles were performed, starting at 20 °C and subsequent heating to 200 °C. A heating rate of 10 K $min^{-1}$ and a nitrogen flow of 40 mL $min^{-1}$ were applied. The analysis of data was performed with the software NETZSCH Proteus – Thermal Analysis.

## 1.5. General synthesis information

The chemicals used were commercially available from Acros Organics, Sigma Aldrich GmbH, TCI GmbH, and Alfa Aesar and used as received. Solvents and the alcohols used were dried if needed according to literature (Table S1). The used polymers were provided by bio-mi Ltd. (Croatia) (polylactide (PLA)) and AIMPLAS (Spain) (polyethylene terephthalate (PET), PET waste material). Further, commercially available poly-L-lactide (PLLA) cups (Ilip Bio, Italy, product code: 61850 - LI20050TCE) were used. PLA film ($M_n$ = 50.8 kg $mol^{-1}$) was cut into small pieces (5 x 5 mm) and stored in a heating oven (50 °C). PLA powder ($M_n$ = 56.6 kg $mol^{-1}$) was cryo-milled to 0.75 mm by the Leibniz Institute for Natural Product Research and Infection Biology - Hans Knöll Institute (HKI, Germany). The bisguanidine organocatalysts were synthesized according to literature.[5-7] The tetramethylguandinium chloride (TMG Vilsmeier salt) was provided and synthesized by Prof. Dr. Sonja Herres-Pawlis and Dr. Alexander Hoffmann after literature procedures and stored under nitrogen atmosphere.[8, 9] Bis(*N*,*N*,*N*,*N*-tetramethylguanidino)-propane ($TMG_2$p or btmgp) was provided by the working group of Prof. Dr. Sonja Herres-Pawlis. All experiments for the determination of $k_{dp}$ and the Eyring and Arrhenius plots were conducted using standard Glovebox and Schlenk technique under inert $N_2$ or Ar atmosphere.

Table S1: Supplier and purities of the used nucleophiles and solvents.

| **Chemical** | **Supplier** | **Purity** |
|---|---|---|
| Acetonirile | Chemsolute | 99.9% |
| Acetonirile-$d_3$ | Eurisotop | 99.8 atom% D |
| $CDCl_3$ | Acros Organics | 99.8%, extra dry over molecular sieve |
| Charcoal | Grüssing | |
| Celite® **535** | Roth | |
| Dimethyl sulfoxide-$d_6$ (DMSO-$d_6$) | Sigma Aldrich | 99.9 atom% D |
| Dichloromethane (DCM) | Fisher Scientific | Synthesis grade |
| Ethylene glycol (EG) | Alfa Aesar | 99% |
| EtOH | Acros Organics | 99.5%, extra dry over molecular sieve |
| EtOH | VWR Chemicals | Analytic reagent grade |
| HCl | Fluka | ≥37% |
| n-hexane | VWR Chemicals | Analytic reagent grade |
| KOH | Fisher Scientific | Analytic reagent grade |
| MeOH | Acros Organics | 99.8%, extra dry over molecular sieve |
| MeOH | Fisher Scientific | Synthesis grade |
| NaOH | Fisher Scientific | Analytic reagent grade |
| $NaSO_4$ | VWR Chemicals | Analytic reagent grade |
| Pentane-1,5-diamine | Fisher Scientific | 98% |
| Polycaprolactone (PCL) | Sigma Aldrich | |
| 1,1,3,3-tetramethylguanidine (TMG) | abcr | 99% |
| Tetrahydrofuran (THF) | Chemsolute | Distilled, degassed, and stored under Ar |
| THF | Fisher Scientific | Synthesis grade |
| Triethyl amine | Sigma Aldrich | ≥99% |

## 2. Evaluation of the depolymerizations of polyesters

The conducted experiments are described in detail in the experimental section in the main article or are available *via* the Chemotion repository including the obtained data and additional information(https://dx.doi.org/10.14272/collection/LB_2024-12-11). The obtained $^1$H NMR spectra for the evaluation of $k_{dp}$, the Eyring plots, and the Arrhenius plots are available *via* the repository RADAR4Chem (https://www.radar-service.eu/radar/en/dataset/2ejvm338fzgnukrr?token=bXMUnNhzOYODdRarjKwg).

### 2.1. The alcoholysis of PLA

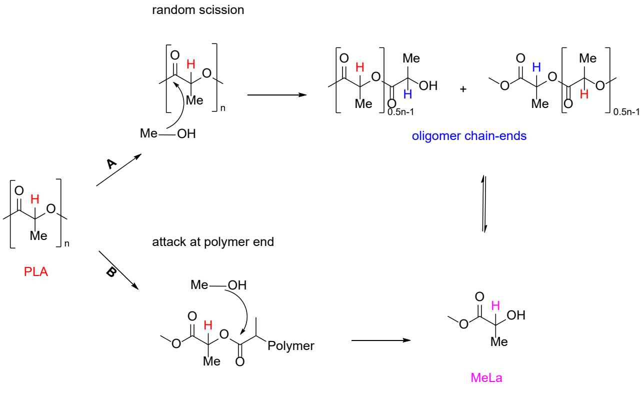


Scheme S1. Two proposed pathways for the alcoholysis of PLA using MeOH as described in literature.[10]

The conversion of PLA follows a *pseudo*-first order mechanism. Monitoring the change of concentration of the internal methine groups ([Int]) as well as the methine group of the oligomers [oligomer] and RLa [RLa] *via* $^1$H NMR spectroscopy allows the determination of the conversion of the internal methine groups of PLA $X_{int}$, the selectivity towards the alkyl lactate $S_{RLa}$, and the yield of RLa $Y_{RLa}$.[11] In Figure S2, an exemplary $^1$H NMR spectrum for the methanolysis is displayed and in Figure S3, an exemplary $^1$H NMR spectrum for the ethanolysis. We use the integral

for the resonances of the internal methine groups of PLA and the oligomers ($I_{int,1}$, 5.15–5.04 ppm), the integral of the resonance of the oligomer end group ($I_{oligomer}$, 4.29–4.23), and the integral of the resonance of RLa ($I_{MeLa}$, 4.22–4.17 ppm, respective $I_{EtLa,1}$, 4.16–4-03 ppm) to calculate the relative concentration of the species. To calculate $I_{int,2}$ the integral only of the resonance for internal methine groups of PLA equation (1) is used.

$$I_{int,2} = I_{int,1} - I_{oligomer} \tag{1}$$

[Int] is calculated using equation (2). [oligomer] and [RLa] are calculated accordingly.

$$[I_{int}] = \frac{I_{int}}{I_{int,2}+I_{oligomer}+I_{RLa}} \tag{2}$$

For the ethanolysis, the resonance for the methine signal of EtLa in the $^1$H NMR spectrum in $CDCl_3$ is overlapped by the $CH_2$-group of the EtOH residue of the oligomer and EtLa. $I_{EtLa,1}$ is set as 1 and $I_{EtLa,2}$ can be calculated according to equation (3).[12]

$$I_{EtLa,2} = \frac{I_{EtLa,1}-2 \cdot I_{oligomer}}{3} \tag{3}$$

All kinetic investigation were performed under standard reaction conditions using PLA (250 mg, 3.47 mmol, 1.00 equiv) dissolved in tetrahydrofuran (THF (4 mL)) at 60 °C (stirring speed = 260 rpm). PLA and catalyst were dissolved prior to the start of the reaction to secure the homogeneity of the reaction mixture from the start.

$$X_{\mathrm{Int}} = \frac{[\mathrm{Int}]_0-[\mathrm{Int}]}{[\mathrm{Int}]_0} \tag{4}$$

$$S_{\mathrm{RLa}} = \frac{[\mathrm{RLa}]}{[\mathrm{Int}]_0-[\mathrm{Int}]} \tag{5}$$

$$Y_{\mathrm{RLa}} = S_{\mathrm{RLa}} \cdot X_{\mathrm{Int}} \tag{6}$$

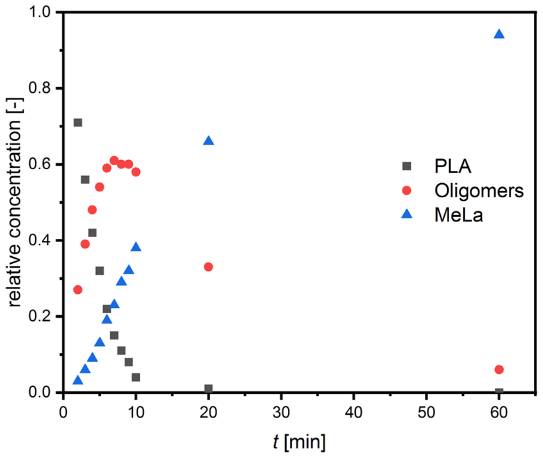


Figure S1. Plot of the relative concentration of PLA, oligomers, and MeLa against time for the methanolysis of PLA (250 mg, 3.47 mmol, 1.00 equiv) using $TMG_2e$ (0.5 mol%, regarding the polymer ester bonds) and MeOH (1.00 mL, 24.7 mmol, 7.13 equiv) in THF (4 mL) at 60 °C and 260 rpm.

### 2.2. Evaluation of the methanolysis of PLA

The methanolysis of PLA was conducted and analyzed according to literature.[12-14] The obtained FAIR data are available *via* the repository RADAR4Chem. Figure S2 exemplarily shows an analyzed $^1$H NMR spectrum for the methanolysis of PLA.

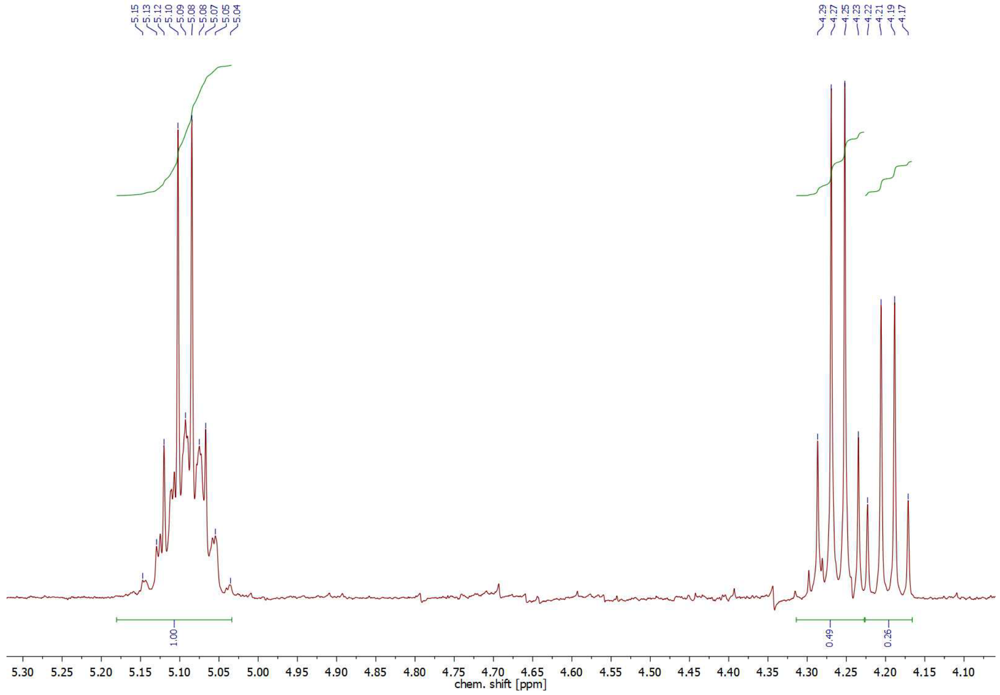


Figure S2. Exemplary $^1$H NMR spectrum of the methanolysis of PLA after 5 min. Reaction conditions: 250 mg PLA, 0.5 mol% $TMG_2e$, 7.1 equiv MeOH, 4 mL THF, 260 rpm, 60 °C.

### 2.3. $TMG_2e$ and $TMG_2p$ vs. $[FeCl_2(TMG_2e)]$ and $[FeCl_2(TMG_2p)]$

Table S2. Comparison of the methanolysis (1 mL, 7.12 equiv) of PLA (250 mg, 3.47 mmol) in THF (4 mL) using 0.5 mol% organocatalysts $TMG_2e$ and $TMG_2p$ with their corresponding Fe(II) complexes (1.0 mol%) at 60 °C and 260 rpm.

| catalyst | *t* [min] | $X_{int}{}^a$ [%] | $S_{MeLa}{}^a$ [%] | $Y_{MeLa}{}^a$ [%] | doi |
|---|---|---|---|---|---|
| $TMG_2e$ | 4 | 58 | 16 | 9 | RADAR4Chem |
| $TMG_2e^b$ | 10 | 100 | 91 | 91 | 1. Experiment: 10.14272/reaction/SA-FUHFF-UHFFFADPSC-LPEKGGXMPW-UHFFFADPSC-NUHFF-NUHFF-NUHFF-ZZZ.10 |
| | 60 | 100±0 | 99±1 | 99±1 | 2. Experiment: 10.14272/reaction/SA-FUHFF-UHFFFADPSC-LPEKGGXMPW-UHFFFADPSC-NUHFF-NUHFF-NUHFF-ZZZ.5 |
| | | | | | 3. Experiment: 10.14272/reaction/SA-FUHFF-UHFFFADPSC-LPEKGGXMPW-UHFFFADPSC-NUHFF-NUHFF-NUHFF-ZZZ.1 |
| $[FeCl_2(TMG_2e)]$[15] | 60 | 54 | 27 | 15 | 10.1039/D3CY01117H |
| $TMG_2p$ | 6 | 57 | 16 | 9 | RADAR4Chem |
| $TMG_2p^b$ | 40 | 100 | 100 | 100 | 10.14272/reaction/SA-FUHFF-UHFFFADPSC-LPEKGGXMPW-UHFFFADPSC-NUHFF-NUHFF-NUHFF-ZZZ.9 |
| $[FeCl_2(TMG_2p)]$[15] | 60 | 52 | 32 | 17 | 10.1039/D3CY01117H |

**a** The conversion of internal methine groups of PLA ($X_{int}$), the selectivity for the alkyl lactate ($S_{RLa}$), and the yield of the alkyl lactate ($Y_{RLa}$) were calculated from $^1$H NMR spectroscopic analysis according to literature.[13] **b** 1.0 mol% catalyst.

## 2.4. Results for the investigation of the influence of the linker length

Table S3. List of experiments corresponding to Table 1 and the corresponding FAIR data.

| Catalyst | # $CH_2$[a] | cat. loading [mol%][b] | *t* [min] | $X_{int}$[c] [%] | $S_{MeLa}$[c] [%] | $Y_{MeLa}$[c] [%] | doi |
|---|---|---|---|---|---|---|---|
| $TMG_2e$ | 2 | 0.5 | 10 | 98 | 46 | 45 | RADAR4Chem |
| $TMG_2e$[c] | 2 | 1.0 | 3 | 99 | 51 | 51 | RADAR4Chem |
| $TMG_2p$ | 3 | 0.5 | 15 | 99 | 51 | 50 | RADAR4Chem |
| $TMG_2p$[c] | 3 | 1.0 | 5 | 98 | 59 | 59 | RADAR4Chem |
| | | | 40 | 100 | 100 | 100 | 10.14272/reaction/SA-FUHFF-UHFFFADPSC-LPEKGGXMPW-UHFFFADPSC-NUHFF-NUHFF-ZZZ.9 |
| $TMG_2pe$ | 5 | 0.5 | 20 | 100 | 61 | 61 | RADAR4Chem |
| TMG | - | 0.5 | 180 | 66 | 19 | 12 | RADAR4Chem |
| TMG[c] | - | 1.0 | 180 | 98±0 | 42±2 | 41±2 | 1.Experiment: 10.14272/reaction/SA-FUHFF-UHFFFADPSC-LPEKGGXMPW-UHFFFADPSC-NUHFF-NUHFF-NUHFF-ZZZ.3<br>2.Experiment: 10.14272/reaction/SA-FUHFF-UHFFFADPSC-LPEKGGXMPW-UHFFFADPSC-NUHFF-NUHFF-NUHFF-ZZZ.6 |
| No catalyst[d] | - | - | 1320 | 0 | 0 | 0 | 10.14272/reaction/SA-FUHFF-UHFFFADPSC-LPEKGGXMPW-UHFFFADPSC-NUHFF-NUHFF-ZZZ.15 |

**a** Number of $CH_2$ groups of the linker between the guanidine functionalities. **b** Catalyst loading in regards to ester bonds in the used PLA (250 mg, 3.47 mmol, 1.00 equiv). **c** $X_{int}$, $S_{RLa}$, and $Y_{RLa}$ were calculated from $^1H$ NMR spectroscopic analysis according to literature.[13] **d** No catalyst was used. See also Section S6.1.

## 2.5. Methanolysis using $TMG_2eH_2Cl_2$

PLA (250 mg, 3.47 mmol, 1.00 equiv), $TMG_2e$ hydrochloride ($TMG_2eH_2Cl_2$, 11.4 mg, 34.6 µmol, 0.001 equiv), and THF (4.00 mL) were provided in a Young-type Schlenk tube. After the PLA film had been dissolved using an external heat source, the tube was placed in a preheated oil bath at 60 °C. MeOH (792 mg, 1.00 mL, 24.7 mmol, 7.13 equiv) was added to start the methanolysis. After 1 h, no PLA conversion was observed in the $^1H$ NMR spectroscopic analysis. The obtained FAIR data is available under doi: 10.14272/reaction/SA-FUHFF-UHFFFADPSC-LPEKGGXMPW-UHFFFADPSC-NUHFF-NUHFF-NUHFF-ZZZ.11.

## 2.6. Methanolysis using $TMG_2e$ and $TMG_2eH_2Cl_2$ (1:1)

PLA (250 mg, 3.47 mmol, 1.00 equiv), $TMG_2e$ hydrochloride ($TMG_2eH_2Cl_2$, 11.4 mg, 34.6 µmol, 0.00998 equiv), and $TMG_2e$ (8.90 mg, 34.7 µmol, 0.0100 equiv) were dissolved in THF (4 mL) using an external heat source in a Young-type Schlenk tube. The reaction was placed in an oil bath at 60 °C and stirred at 260 rpm. To start the reaction MeOH (792 mg, 1.00 mL, 24.7 mmol, 7.12 equiv) was added to start the reaction. $^1H$ NMR samples were taken after 2-10 min, 15 min, 30 min, and 60 min. After 60 min, 99% conversion of PLA was observed ($Y_{MeLa}$ = 68%). Complete selective conversion of PLA to MeLa was observed after 150 min ($Y_{MeLa}$ = 100%). The reaction was stopped after 21 h, oligomers had formed again in an equilibrium reaction with the alkyl lactate ($S_{MeLa}$ = 94%). The obtained FAIR data is available under doi: 10.14272/reaction/SA-FUHFF-UHFFFADPSC-LPEKGGXMPW-UHFFFADPSC-NUHFF-NUHFF-NUHFF-ZZZ.7.

Table S4. List of experiments performed using $TMG_2eH_2Cl_2$ (1 mol%) with or without addition of $TMG_2e$.

| Ratio $TMG_2eH_2Cl_2$ : $TMG_2e$ | *t* [min] | $X_{int}$[a] [%] | $S_{MeLa}$[a] [%] | $Y_{MeLa}$[a] [%] | $k_{app}$ [$min^{-1}$] | doi |
|---|---|---|---|---|---|---|
| 1:0 | 60 | 0 | 0 | 0 | - | RADAR4Chem |
| | 4320 | 2 | 0 | 0 | | |
| 1:1 | 5 | 21 | 4 | 1 | 0.067 | RADAR4Chem |
| | 60 | 99 | 69 | 68 | | |

**a** $X_{int}$, $S_{RLa}$, and $Y_{RLa}$ were calculated from $^1H$ NMR spectroscopic analysis according to literature.[13]

### 2.7. Evaluation of the ethanolysis of PLA

The ethanolysis of PLA was analyzed according to literature.[12-14] The obtained FAIR data is available in the repository RADAR4Chem. Figure S3 exemplarily shows an analyzed $^1$H NMR spectrum for the ethanolysis of PLA.

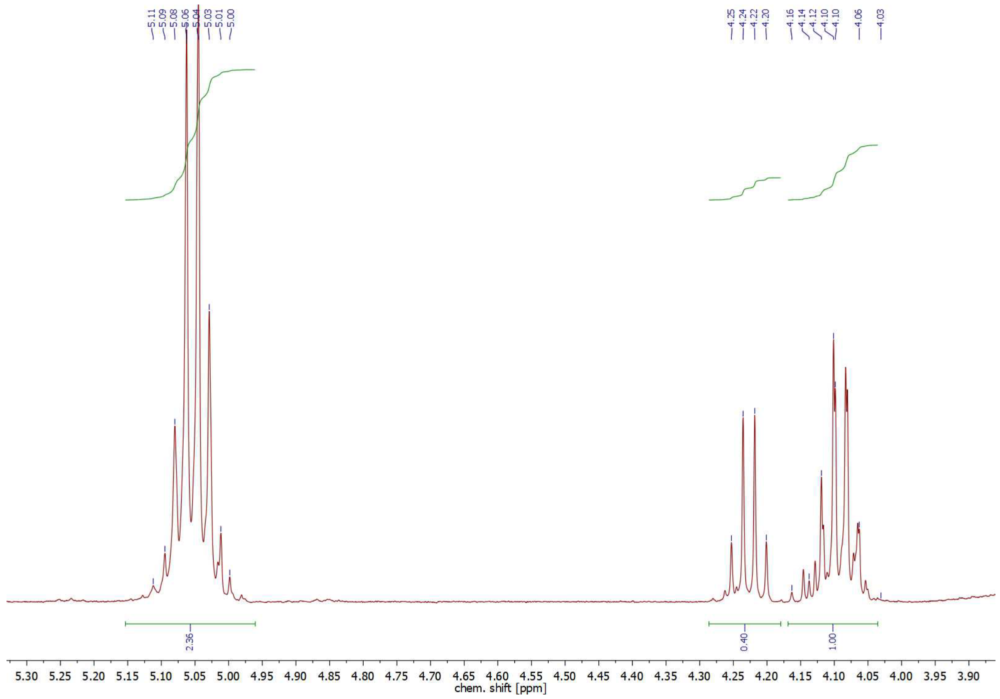


Figure S3. Exemplary $^1$H NMR spectrum of the ethanolysis of PLA after 120 min. Reaction conditions: 250 mg PLA, 0.5 mol% $TMG_2e$, 6.9 equiv EtOH, 4 mL THF, 260 rpm, 60 °C.

### 2.8. Evaluation of the methanolysis of PCL

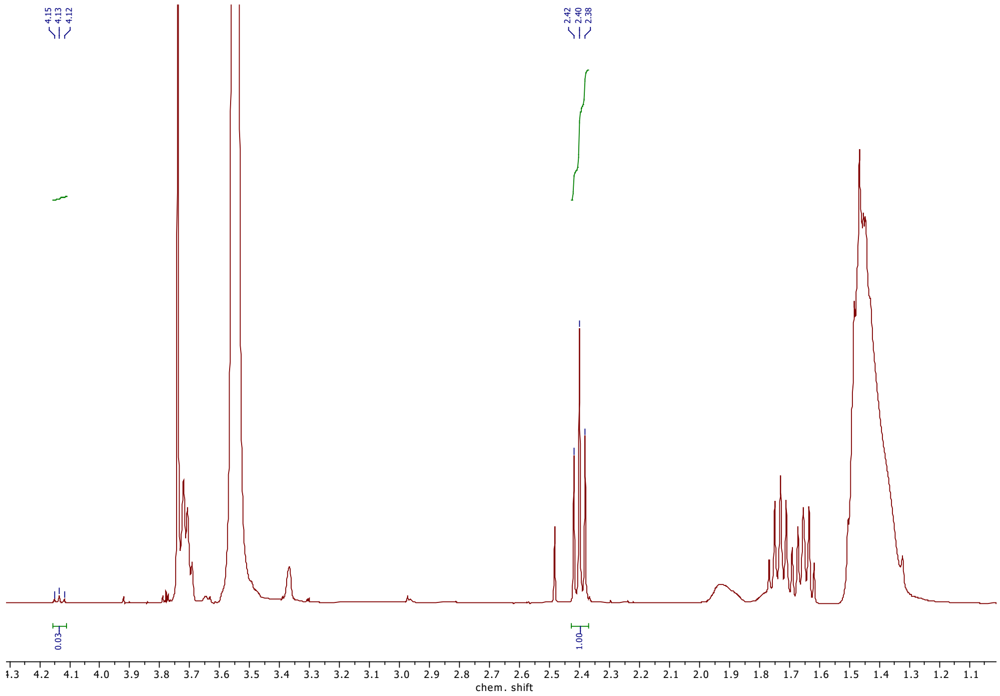


Figure S4. Exemplary $^1$H NMR spectrum of the methanolysis of PCL after 60 min. Reaction conditions: 400 mg PCL, 2 mol% $TMG_2e$, 28 equiv MeOH, 260 rpm, 100 °C.

The methanolysis of PCL was conducted and analyzed according to literature.[16] The yield of methyl 6-hydroxyhexanoate (MeHe) was determined using the integral at 4.15–4.13 ppm (**I**$_A$) and 2.42–2.38 ppm (**I**$_B$) in equation (7).

$$Y_{MeHe} = \frac{I_B - I_A}{I_B} \cdot 100\% \tag{7}$$

The obtained FAIR data for the experiment in Table 5 is available under doi: 10.14272/reaction/SA-FUHFF-UHFFFADPSC-YDJZXHZRXD-UHFFFADPSC-NUHFF-NUHFF-NUHFF-ZZZ.1 and doi: 10.14272/reaction/SA-FUHFF-UHFFFADPSC-YDJZXHZRXD-UHFFFADPSC-NUHFF-NUHFF-NUHFF-ZZZ.2.

### 2.9. Methanolysis of PCL in presence of MeLa

PCL (397 mg, 3.48 mmol, 1.00 equiv), MeLa (361 mg, 330 µL, 3.46 mmol, 0.995 equiv), $TMG_2e$ (17.8 mg, 69.4 µmol, 0.0199 equiv), and MeOH (3.17 g, 4.00 mL, 98.9 mmol, 28.4 equiv) were provided in a Young-type Schlenk tube. The tube was placed in a preheated oil bath at 100 °C. After 60 min, only resonances for PCL could be assigned in the $^1H$ NMR spectrum. No depolymerization activity could be observed. The obtained FAIR data is available under doi: 10.14272/reaction/SA-FUHFF-UHFFFADPSC-YDJZXHZRXD-UHFFFADPSC-NUHFF-NUHFF-NUHFF-ZZZ.

### 2.10. Evaluation of the methanolysis of PET

The methanolysis of PET was conducted and analyzed according to literature.[17, 18] The obtained FAIR data for the experiment in Table 5 is available under doi: 10.14272/reaction/SA-FUHFF-UHFFFADPSC-WOZVHXUHUF-UHFFFADPSC-NUHFF-NUHFF-NUHFF-ZZZ.1.

### 2.11. Evaluation of the glycolysis of PET

The glycolysis of PET was conducted and analyzed according to literature.[17-19] The obtained FAIR data for the experiment in Table 5 are provided in Table S4.

Table S5. Link to the obtained FAIR data for the performed glycolysis experiments listed in Table 5.

| polymer | $m_{polymer}$ [g] | Nu | doi |
|---|---|---|---|
| PET | 0.67 | EG | 10.14272/reaction/SA-FUHFF-UHFFFADPSC-QPKOBORKPH-UHFFFADPSC-NUHFF-NUHFF-NUHFF-ZZZ.4 |
| PET | 3.0 | EG | 10.14272/reaction/SA-FUHFF-UHFFFADPSC-QPKOBORKPH-UHFFFADPSC-NUHFF-NUHFF-NUHFF-ZZZ |

### 2.12. Control reaction using EG and $TMG_2e$

$TMG_2e$ (266 mg, 1.04 mmol, 0.0200 equiv) were provided in a Schlenk tube. EG (22.3 g, 20.0 mL, 359 mmol, 6.90 equiv) was added. The mixture was placed in an oil bath. The reaction was stirred with 300 rpm at 180 °C for 48 h. After 48 h, the reaction was allowed to cool to room temperature. $^1H$ NMR samples were taken after 24 h and 48 h. $^1H$ NMR spectroscopic analysis showed no catalytic activity. The obtained FAIR data is available under doi: 10.14272/reaction/SA-FUHFF-UHFFFADPSC-LYCAIKOWRP-UHFFFADPSC-NUHFF-NUHFF-NUHFF-ZZZ.

### 2.13. Control reactions using BHET and THF

Bis(2-hydroxyethyl) benzene-1,4-dicarboxylate (BHET, 52.0 mg, 205 µmol, 1.00 equiv) and THF (0.25 mL) were provided in a Young-type Schlenk flask. The reaction was placed in an oil bath and stirred at 260 rpm. The oil bath was heated to 180 °C within 68 min. After 1 h at 180 °C, no visible change was observed. After 5 h at 180 °C, the reaction mixture had turned dark brown. The reaction mixture was cooled to room temperature and analyzed using $^1H$ NMR spectroscopy. High formation of other species was observed. The obtained FAIR data is available under doi: 10.14272/reaction/SA-FUHFF-UHFFFADPSC-QPKOBORKPH-UHFFFADPSC-NUHFF-NUHFF-NUHFF-ZZZ.11.

### 2.14. Control reactions using BHET, $TMG_2e$, and THF

$TMG_2e$ (4.10 mg, 16.0 µmol, 0.0203 equiv), BHET (200 mg, 787 µmol, 1.00 equiv), and THF (1.00 mL) were provided in a Young-type Schlenk flask. The reaction was placed in an oil bath and stirred at 260 rpm. The oil bath was heated to 180 °C within 68 min. After 1 h at 180 °C, the reaction mixture had turned from light brown to dark brown. After 5 h, the reaction mixture was cooled to room temperature and analyzed using $^1H$ NMR spectroscopy. High formation of other species was observed. The obtained FAIR data is available under doi: 10.14272/reaction/SA-FUHFF-UHFFFADPSC-QPKOBORKPH-UHFFFADPSC-NUHFF-NUHFF-NUHFF-ZZZ.10.

### 2.15. Control reactions using BHET, $TMG_2e$, and EG

$TMG_2e$ (3.90 mg, 15.2 µmol, 0.0193 equiv), BHET (200 mg, 787 µmol, 1.00 equiv), and EG (1.11 g, 1.00 mL, 17.9 mmol, 22.7 equiv) were provided in a Young-type Schlenk flask. The reaction was placed in an oil bath and stirred at 260 rpm. The oil bath was heated to 180 °C within 68 min. After 1 h at 180 °C, no visible change was observed. After 3 h at 180 °C, the reaction mixture turned darker brown. After 5 h at 180 °C, the reaction mixture had turned dark brown. The reaction mixture was cooled to room temperature and analyzed using $^1H$ NMR spectroscopy. Low formation of other species was observed. The obtained FAIR data is available under doi: 10.14272/reaction/SA-FUHFF-UHFFFADPSC-QPKOBORKPH-UHFFFADPSC-NUHFF-NUHFF-NUHFF-ZZZ.12.

# 3. Determination of $k_{dp}$ for the methanolysis and ethanolysis of PLA

For the determination of the reaction rate constant $k_{dp}$ the conversion of PLA is determined according to literature.[12-14] The change of the concentration of the internal methine group of PLA ([Int]) is monitored using $^1$H NMR spectroscopy. First, the apparent reaction rate constant $k_{app}$ is obtained as the slope of the semi-logarithmic plot of $\ln([Int]_0) - \ln([Int]_t)$ versus $t$. For reactions following a pseudo-first order mechanism a linear regression is obtained. The depolymerization rate $k_{dp}$ is determined by plotting $k_{app}$ versus the catalyst loading ($c$):[12, 14] The obtained data is published in the repository RADAR4Chem.

$$[Int]_0 = [Int]_t + [oligomers] + [RLa] \tag{8}$$

$$k_{dp} = \frac{k_{app}}{c} \tag{9}$$

### 3.1. Determination of $k_{dp}$ for the methanolysis of PLA using TMG$_2$e as catalyst

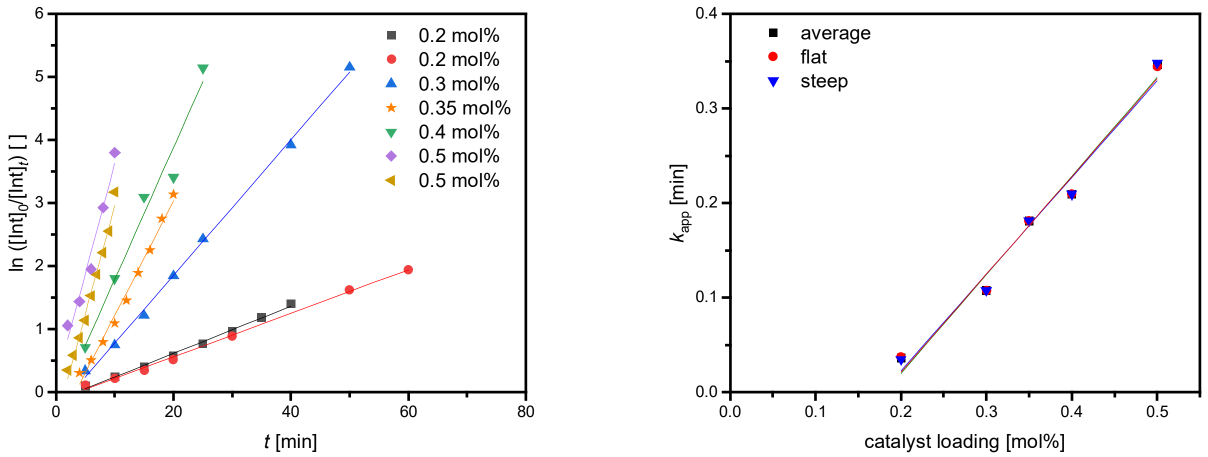


Figure S5. Left: Semilogarithmic plots of ln([Int]$_0$/[Int]$_t$) against time for the alcoholysis of PLA (250 mg, 3.47 mmol, 1.00 equiv) for different TMG$_2$e concentrations (0.2, 0.3, 0.35, 0.4, 0.5 mol%, regarding the polymer ester bonds) using MeOH (1.00 mL, 24.7 mmol, 7.13 equiv) in THF (4 mL) at 60 °C and 260 rpm. Right: Determination of $k_{dp}$ and the error of $k_{dp}$.

### 3.2. Determination of $k_{dp}$ for the methanolysis of PLA using TMG$_2$p as catalyst

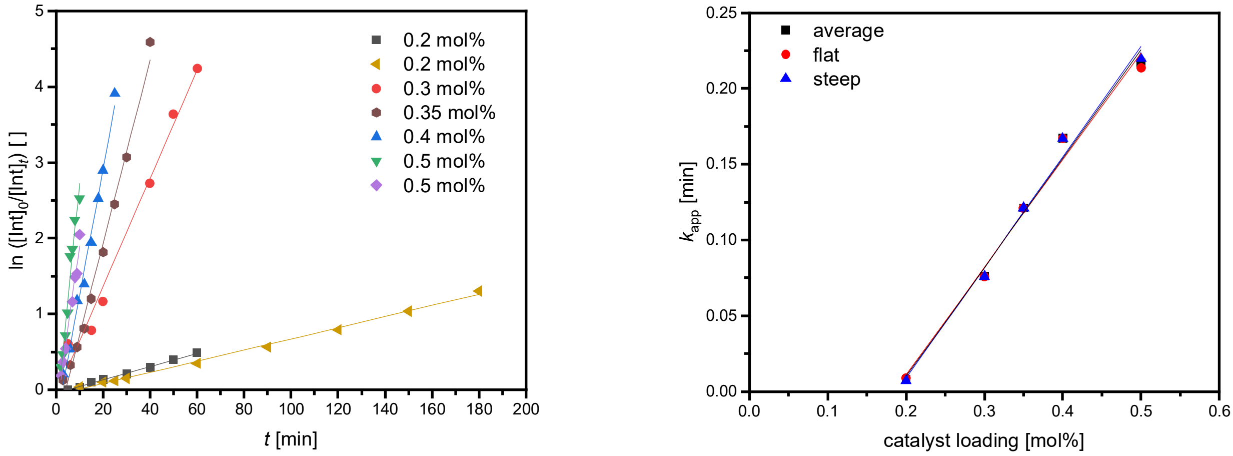


Figure S6. Left: Semilogarithmic plots of ln([Int]$_0$/[Int]$_t$) against time for the alcoholysis of PLA (250 mg, 3.47 mmol, 1.00 equiv) for different TMG$_2$p concentrations (0.2, 0.3, 0.35, 0.4, 0.5 mol%, regarding the polymer ester bonds) using MeOH (1.00 mL, 24.7 mmol, 7.13 equiv) in THF (4 mL) at 60 °C and 260 rpm. Right: Determination of $k_{dp}$ and the error of $k_{dp}$.

## 3.3. Determination of $k_{dp}$ for the methanolysis of PLA using TMG2pe as catalyst

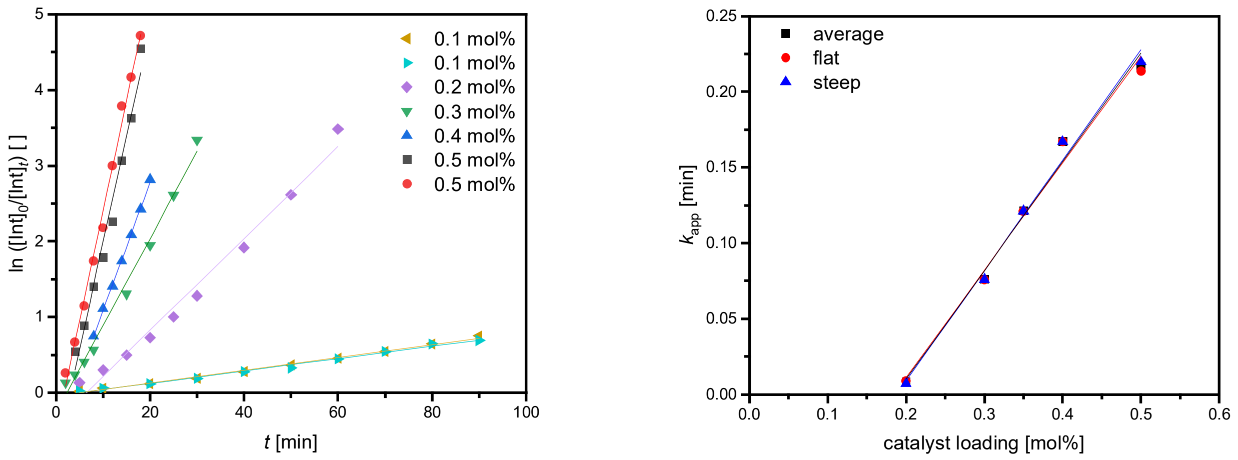


Figure S7. Left: Semilogarithmic plots of ln([Int]$_0$/[Int]$_t$) against time for the alcoholysis of PLA (250 mg, 3.47 mmol, 1.00 equiv) for different TMG$_2$pe concentrations (0.1, 0.2, 0.3, 0.4, 0.5 mol%, regarding the polymer ester bonds) using MeOH (1.00 mL, 24.7 mmol, 7.13 equiv) in THF (4 mL) at 60 °C and 260 rpm. Right: Determination of $k_{dp}$ and the error of $k_{dp}$.

## 3.4. Determination of $k_{dp}$ for the ethanolysis of PLA using TMG$_2$e as catalyst

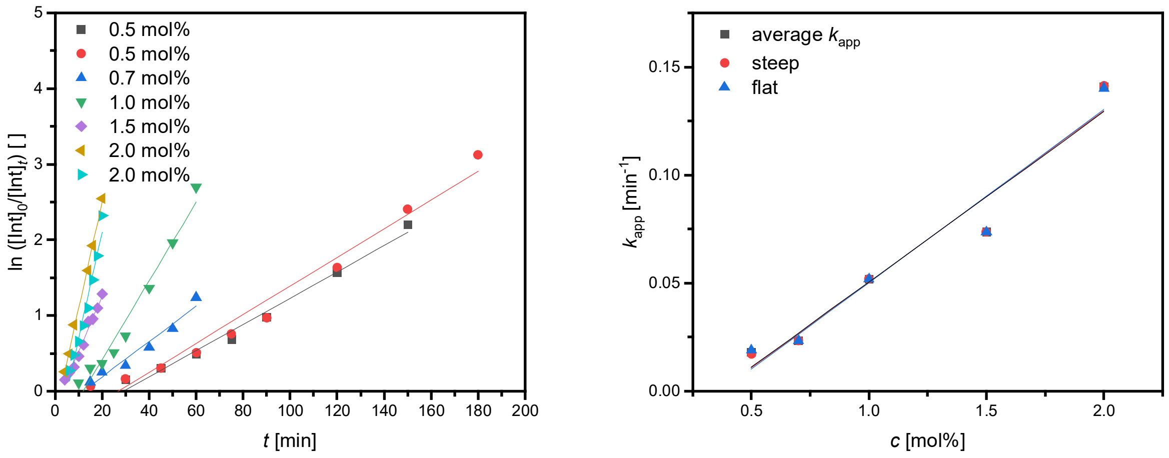


Figure S8. Left: Semilogarithmic plots of ln([Int]$_0$/[Int]$_t$) against time for the alcoholysis of PLA (250 mg, 3.47 mmol, 1.00 equiv) for different TMG$_2$e concentrations (0.5, 0.7, 1.0, 1.5, 2.0 mol%, regarding the polymer ester bonds) using EtOH (1.4 mL, 24 mmol, 6.9 equiv) in THF (4 mL) at 60 °C and 260 rpm. Right: Determination of $k_{dp}$ and the error of $k_{dp}$.

### 3.5. Determination of $k_{dp}$ for the ethanolysis of PLA using $TMG_2p$ as catalyst

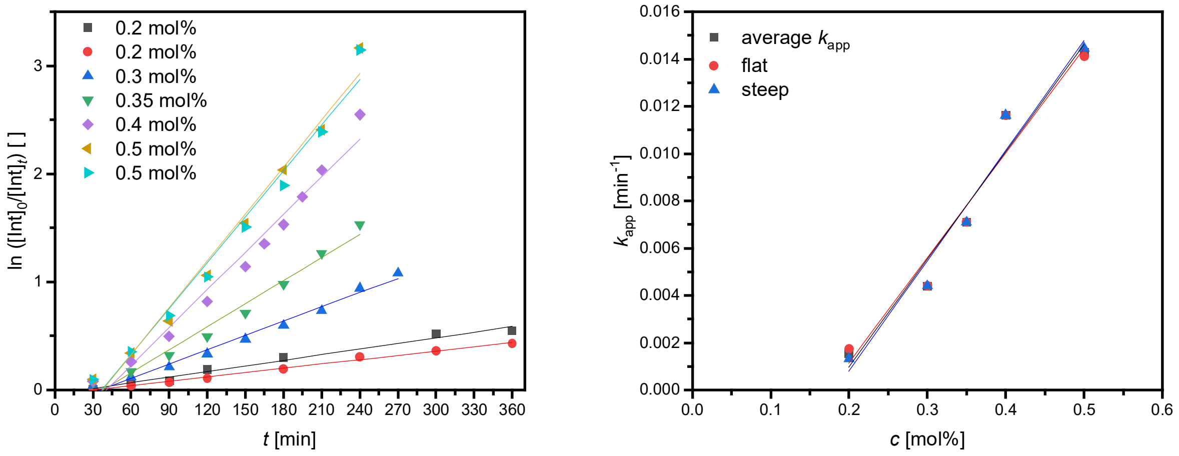


Figure S9. Left: Semilogarithmic plots of ln([Int]₀/[Int]$_t$) against time for the alcoholysis of PLA (250 mg, 3.47 mmol, 1.00 equiv) for different $TMG_2p$ concentrations (0.2, 0.3, 0.35, 0.4, 0.5 mol%, regarding the polymer ester bonds) using EtOH (1.4 mL, 24 mmol, 6.9 equiv) in THF (4 mL) at 60 °C and 260 rpm. Right: Determination of $k_{dp}$ and the error of $k_{dp}$.

# 4. Determination of $E_A$, $\Delta H^{\neq}$ , and $\Delta S^{\neq}$ for the methanolysis and ethanolysis of PLA

Using the Eyring and Arrhenius plot allows the determination of $E_A$, $\Delta H^{\neq}$ , and $\Delta S^{\neq}$ . $E_A$ is determined using the slope $m_A$ of the Arrhenius plot and $\Delta H^{\neq}$ using the slope $m_E$ of the Eyring plot. $\Delta S^{\neq}$ can be calculated using the intercept of the y-axis $y_0$ of the Eyring plot.

$$E_A = -m_A \cdot R \tag{6}$$

$$\Delta H^{\neq} = -m_E \cdot R \tag{7}$$

$$\Delta S^{\neq} = \left(y_0 - ln\left(\frac{k_b}{h}\right)\right) \cdot R \tag{8}$$

The calculations were performed with the Boltzmann constant $k_b$, the Planck constant $h$, and the gas constant $R$. The obtained data is published in the repository RADAR4Chem.

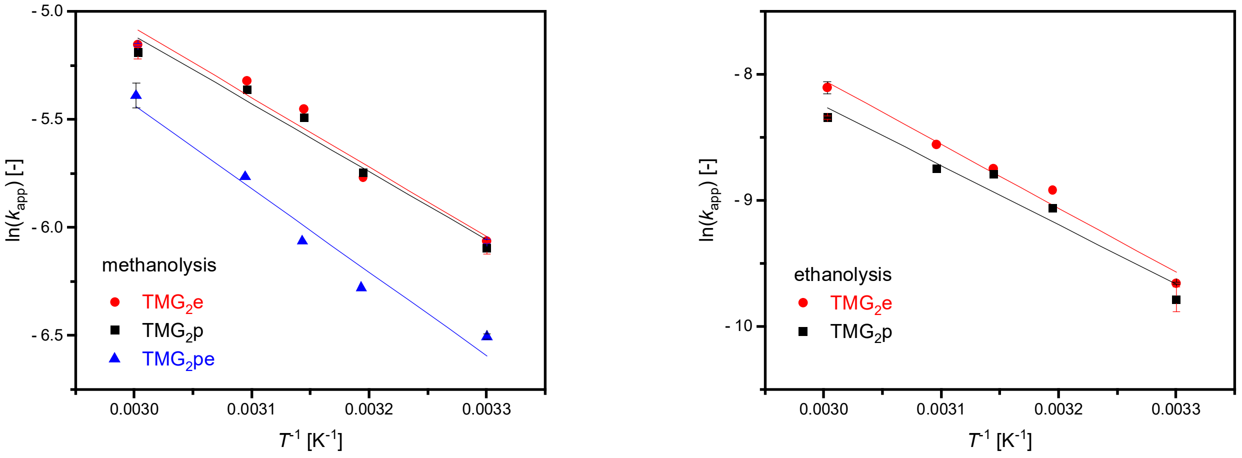


Figure S10. Left: Arrhenius plots for the methanolysis (7.1 equiv) of PLA (250 mg, 3.47 mmol, 1.00 equiv) using 0.5 mol% of $TMG_2e$ (red dots), $TMG_2p$ (black squares), and $TMG_2pe$ (blue triangles) at 30, 35, 40, 45, 50 and 60 °C and 260 rpm. Right: Arrhenius plots for the ethanolysis (6.9 equiv) of PLA (250 mg, 3.47 mmol, 1.00 equiv) using 0.5 mol% of $TMG_2e$ (red dots), and $TMG_2p$ (black squares) at 30, 35, 40, 45, 50 and 60 °C and 260 rpm.

## 4.1. Methanolysis of PLA at different *T* using TMG₂e as catalyst

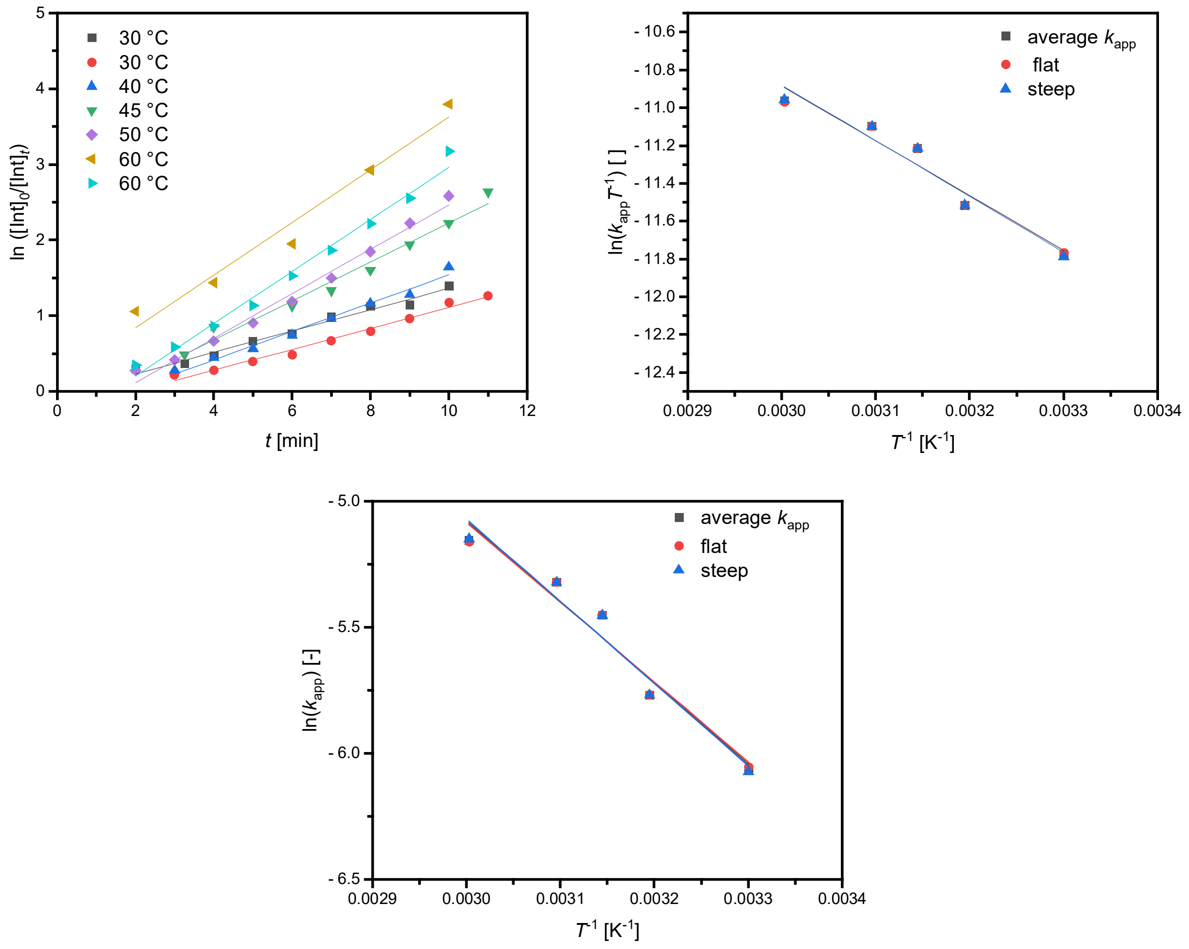


Figure S11. Left: Semilogarithmic plots of ln([Int]$_0$/[Int]$_t$) against time for the alcoholysis of PLA (250 mg, 3.47 mmol, 1.00 equiv) using TMG$_2$e (17.3 µmol, 0.5 mol%, regarding the polymer ester bonds) using MeOH (1.00 mL, 24.7 mmol, 7.13 equiv) in THF (4 mL) at 30, 40, 45, 50 and 60 °C and 260 rpm. Right: Determination of the Δ$H^{\neq}$ and Δ$S^{\neq}$ and the desired errors. Bottom: Determination of $E_A$. and the error of $E_A$.

### 4.2. Methanolysis of PLA at different $T$ using TMG₂p as catalyst

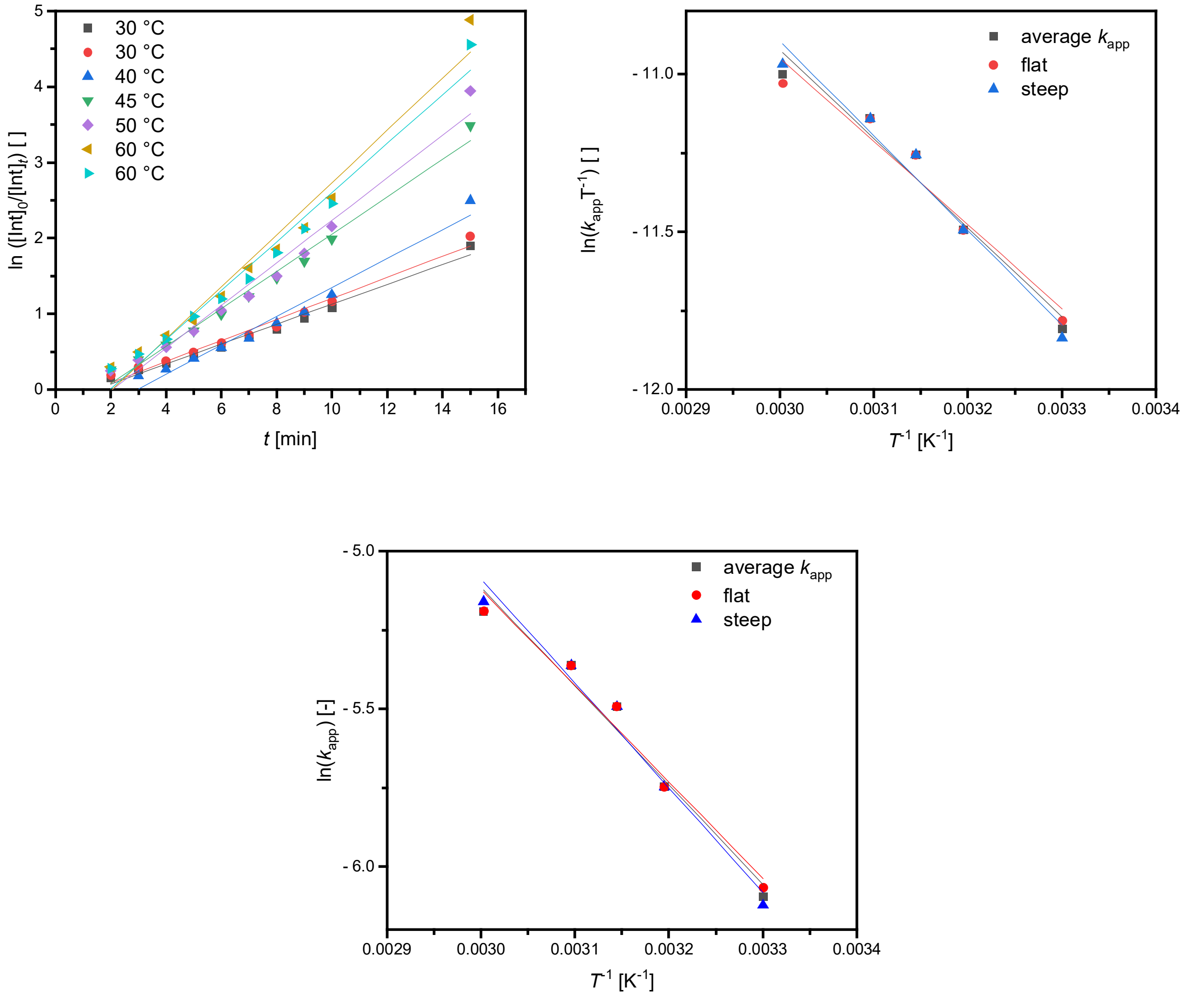


Figure S12. Left: Semilogarithmic plots of ln([Int]$_0$/[Int]$_t$) against time for the alcoholysis of PLA (250 mg, 3.47 mmol, 1.00 equiv) using TMG$_2$e (17.3 µmol, 0.5 mol%, regarding the polymer ester bonds) using MeOH (1.00 mL, 24.7 mmol, 7.13 equiv) in THF (4 mL) at 30, 40, 45, 50 and 60 °C and 260 rpm. Right: Determination of the $\Delta H^{\neq}$ and $\Delta S^{\neq}$ and the desired errors. Bottom: Determination of $E_A$. and the error of $E_A$.

### 4.3. Methanolysis of PLA at different $T$ using TMG₂pe as catalyst

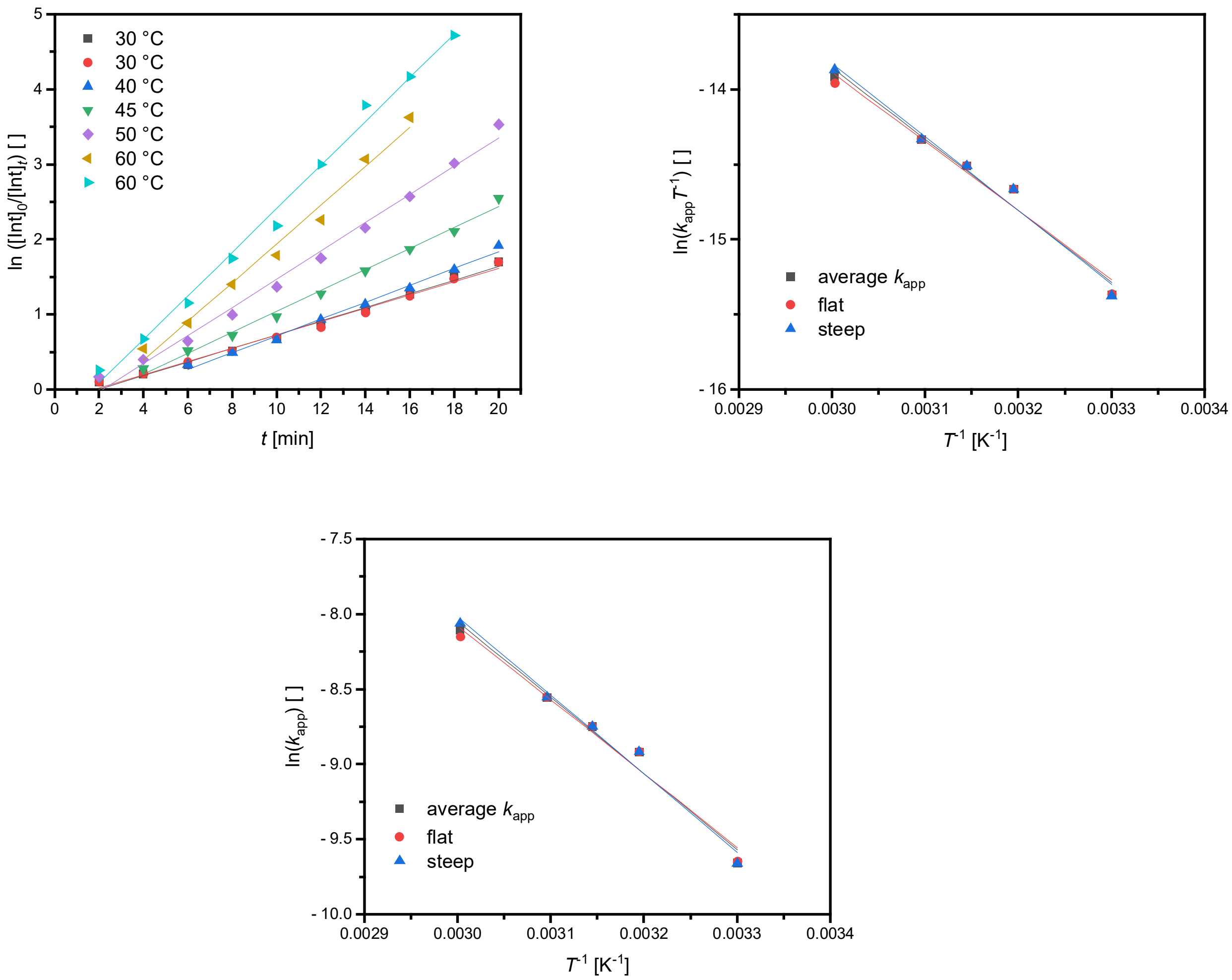


Figure S13. Left: Semilogarithmic plots of ln([Int]$_0$/[Int]$_t$) against time for the alcoholysis of PLA (250 mg, 3.47 mmol, 1.00 equiv) using TMG$_2$e (17.3 µmol, 0.5 mol%, regarding the polymer ester bonds) using MeOH (1.00 mL, 24.7 mmol, 7.13 equiv) in THF (4 mL) at 30, 40, 45, 50 and 60 °C and 260 rpm. Right: Determination of the $\Delta H^{\neq}$ and $\Delta S^{\neq}$ and the desired errors. Bottom: Determination of $E_A$. and the error of $E_A$.

### 4.4. Ethanolysis of PLA at different *T* using TMG$_2$e as catalyst

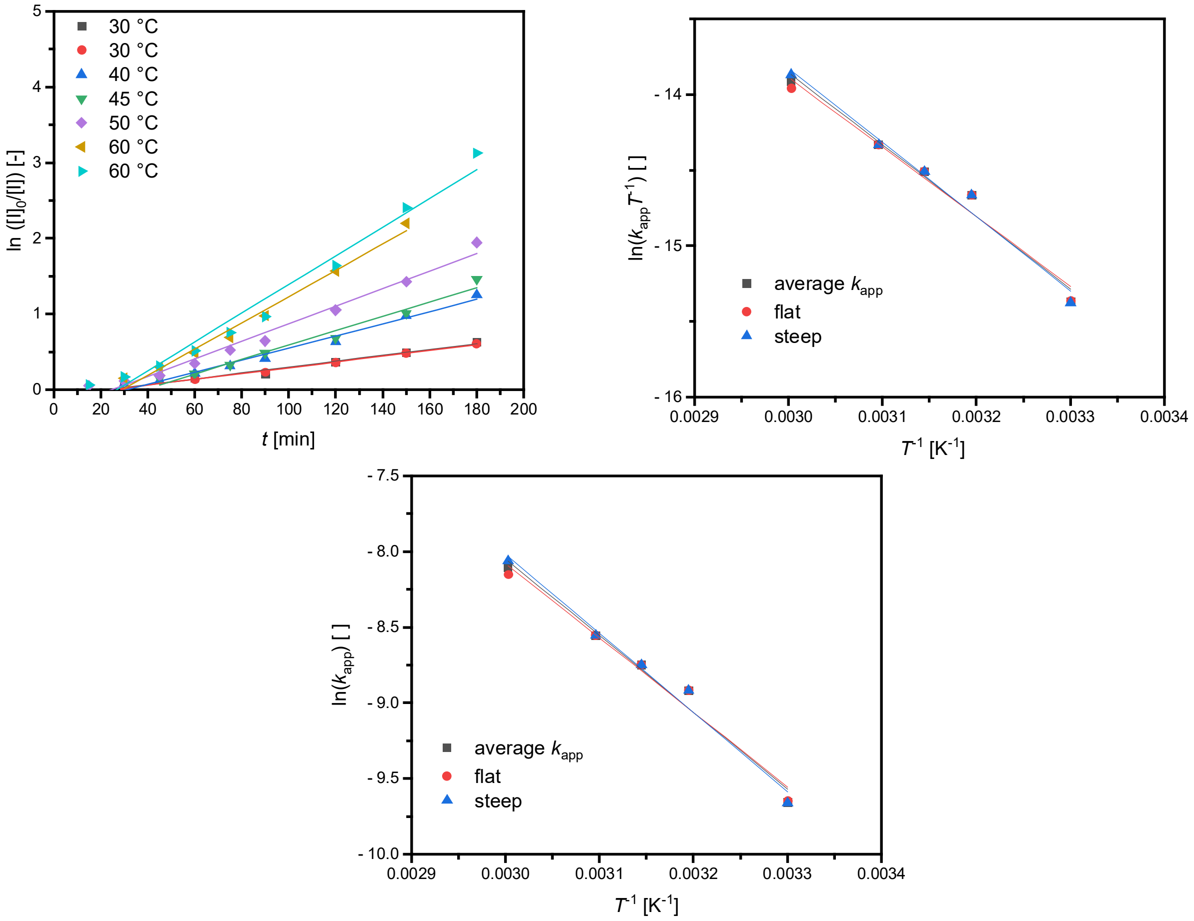


Figure S14. Left: Semilogarithmic plots of ln([Int]$_0$/[Int]$_t$) against time for the alcoholysis of PLA (250 mg, 3.47 mmol, 1.00 equiv) using TMG$_2$e (17.3 µmol, 0.5 mol%, regarding the polymer ester bonds) using EtOH (1.4 mL, 24 mmol, 6.9 equiv) in THF (4 mL) at 30, 40, 45, 50 and 60 °C and 260 rpm. Right: Determination of the $\Delta H^{\neq}$ and $\Delta S^{\neq}$ and the desired errors. Bottom: Determination of $E_A$. and the error of $E_A$.

### 4.5. Ethanolysis of PLA at different *T* using $TMG_2p$ as catalyst

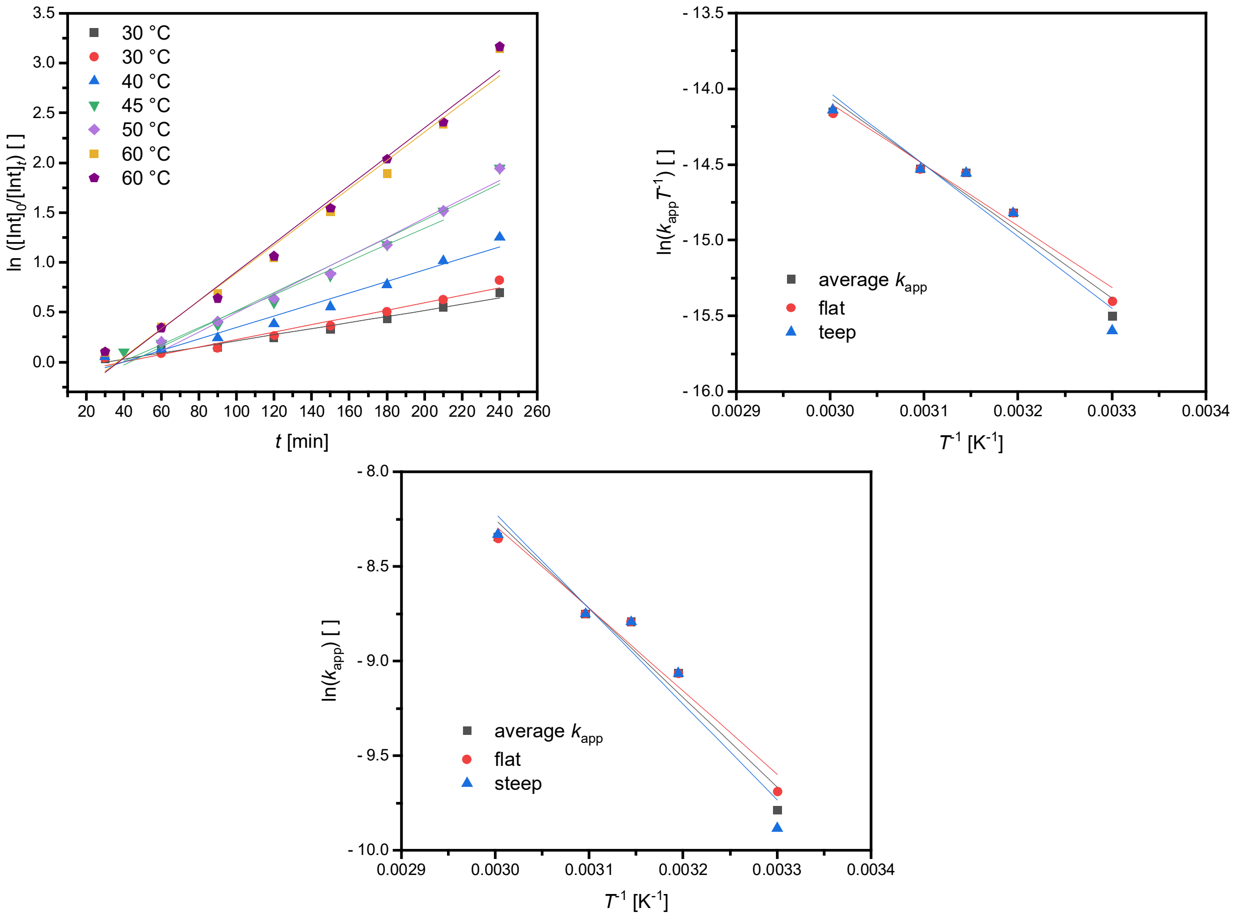


Figure S15. Left: Semilogarithmic plots of ln($[Int]_0/[Int]_t$) against time for the alcoholysis of PLA (250 mg, 3.47 mmol, 1.00 equiv) using $TMG_2e$ (17.3 µmol, 0.5 mol%, regarding the polymer ester bonds) using EtOH (1.4 mL, 24 mmol, 6.9 equiv) in THF (4 mL) at 30, 40, 45, 50, and 60 °C and 260 rpm. Right: Determination of the $\Delta H^{\neq}$ and $\Delta S^{\neq}$ and the desired errors. Bottom: Determination of $E_A$. and the error of $E_A$.

## 5. Determination of $k_{app}$ of the methanolysis using TMG

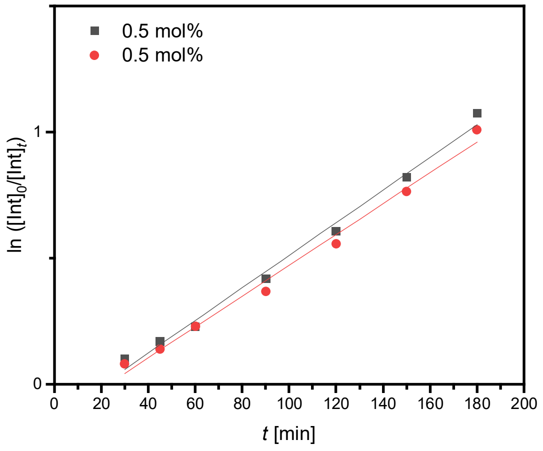


Figure S16. Semilogarithmic plots of ln($[Int]_0/[Int]_t$) against time for the alcoholysis of PLA (250 mg, 3.47 mmol, 1.00 equiv) using 0.5 mol% (regarding the polymer ester bonds) TMG using MeOH (1.00 mL, 24.7 mmol, 7.13 equiv) in THF (4 mL) at 60 °C and 260 rpm.

## 6. Control experiments and solubility behavior

### 6.1. Methanolysis under standard reaction conditions without a catalyst

To confirm that PLA is not depolymerized by MeOH without a catalyst under standard conditions. PLA (250 mg, 3.47 mmol, 1.00 equiv) was dissolved in THF (4 mL). Subsequently, MeOH (792 mg, 1.00 mL, 24.7 mmol, 7.12 equiv) was added. After 22 h, no depolymerization could be observed using $^1H$ NMT spectroscopic analysis. The obtained FAIR data for this experiment is available under doi: 10.14272/reaction/SA-FUHFF-UHFFFADPSC-LPEKGGXMPW-UHFFFADPSC-NUHFF-NUHFF-NUHFF-ZZZ.15.

### 6.2. Control experiment using no catalyst and investigation of the solubility behavior of PLA in EtOH

To confirm that PLA is not depolymerized by EtOH without a catalyst and investigate the solubility behavior of PLA in EtOH, we provided PLA powder (250 mg, 3.47 mmol, 1.00 equiv) and ethanol (1.1 g, 1.4 mL, 24 mmol, 6.9 equiv) in a Young-type Schlenk flask and stirred the mixture 22 h at 100 °C. The plastic swelled and formed a lump around the magnetic stirrer but was clearly visible after 22 h. $^1H$ NMR spectroscopic analysis showed only marginal traces of an oligomeric species of PLA. The obtained FAIR data for this experiment is available under doi: 10.14272/reaction/SA-FUHFF-UHFFFADPSC-LZCLXQDLBQ-UHFFFADPSC-NUHFF-NUHFF-NUHFF-ZZZ.7.

## 7. Large-scale methanolysis and ethanolysis of PLA

The obtained FAIR data for the experiments listed in Table 7 is available under the given dois in Table S6.

Table S6. Link to the obtained FAIR data for the performed large-scale alcoholysis listed in Table 4.

| ROH | $m_{PLA}$ [g] | $t$ [h] | $X_{int}^a$ [%] | $S_{RLa}^a$ [%] | $Y_{RLa}^a$ [%] | $Y_{RLa}^b$ [%] | doi |
|---|---|---|---|---|---|---|---|
| MeOH | 5 | 0.25 | 100 | 100 | 100 | - | 10.14272/reaction/SA-FUHFF-UHFFFADPSC-LPEKGGXMPW-UHFFFADPSC-NUHFF-NUHFF-NUHFF-ZZZ |
| MeOH | 50 | 1.25 | 100 | 100 | 100 | 54 | 10.14272/reaction/SA-FUHFF-UHFFFADPSC-LPEKGGXMPW-UHFFFADPSC-NUHFF-NUHFF-NUHFF-ZZZ.2 |
| EtOH | 5 | 23 | 100 | 100 | 100 | 76 | 10.14272/reaction/SA-FUHFF-UHFFFADPSC-LZCLXQDLBQ-UHFFFADPSC-NUHFF-NUHFF-NUHFF-ZZZ.2 |
| EtOH$^c$ | 175 | 25<br>2 weeks | 100<br>- | 67<br>- | 67<br>- | -<br>71 | 10.14272/reaction/SA-FUHFF-UHFFFADPSC-LZCLXQDLBQ-UHFFFADPSC-NUHFF-NUHFF-NUHFF-ZZZ |
| EtOH$^d$ | 30 | 24 | 95 | 94 | 89 | 65 | 10.14272/reaction/SA-FUHFF-UHFFFADPSC-LZCLXQDLBQ-UHFFFADPSC-NUHFF-NUHFF-NUHFF-ZZZ.1 |
| EtOH | 40 | 2<br>6 | 100<br>- | 89<br>- | 89<br>- | -<br>82 | 10.14272/reaction/SA-FUHFF-UHFFFADPSC-XFEBIZUDWW-UHFFFADPSC-NUHFF-NUHFF-NUHFF-ZZZ |

**a** Determined with $^1H$ NMR spectroscopic analysis and calculated according to literature.[13] Samples were taken from a homogeneous mixture. **b** Isolated yield of RLa. **c** 0.5 mol% $TMG_2e$. **d** Recycled EtOH from the ethanolysis of 175 g PLA was used.

# 8. Depolymerization of mixed plastics

The methanolysis of PET, PCL, and PLA was conducted and analyzed according to literature.[17, 18] The obtained FAIR data for the experiments listed in Table 6 is available under the given dois in Table S7.

Table S7. Results for the methanolysis (4 mL MeOH) of plastic mixes using 2 mol% $TMG_2e$ regarding PLA or PCL ester bonds. PLA (250 mg, 3.5 mmol), PCL (3.4-3.5 mmol), and PET (3.5 mmol) were applied according to the stated content of the polymer mix. If not stated otherwise, PLA film was used. The link to the obtained FAIR data for the performed depolymerization experiments listed in Table 6.

| Polymers | *T* [°C] | *t* [h][a] | $Y_{MeLa}$[b] [%] | $Y_{MeHe}$[c] [%] | $Y_{DMT}$[d] [%] | doi |
|---|---|---|---|---|---|---|
| PLA, PCL | 60 | 1 | 100 | 24 | - | 10.14272/reaction/SA-FUHFF-UHFFFADPSC-FKUNVDJLEY-UHFFFADPSC-NUHFF-NUHFF-NUHFF-ZZZ |
| | 100 | 1 | 100 | 74 | - | |
| | 100 | 20 | 100 | 79 | - | |
| PLA, PET | 60 | 1 | 100 | - | - | 10.14272/reaction/SA-FUHFF-UHFFFADPSC-WPTVLKDYAV-UHFFFADPSC-NUHFF-NUHFF-NUHFF-ZZZ |
| | 180 | 5 | 100 | - | 73 | |
| PCL, PET | 100 | 1 | - | 86 | - | 10.14272/reaction/SA-FUHFF-UHFFFADPSC-MNBJTTBFXG-UHFFFADPSC-NUHFF-NUHFF-NUHFF-ZZZ |
| | 180 | 5 | - | 95 | 80 | |
| PLA, PCL, PET[e] | 60 | 24 | - | - | - | 10.14272/reaction/SA-FUHFF-UHFFFADPSC-QVSUDYMNWS-UHFFFADPSC-NUHFF-NUHFF-NUHFF-ZZZ.4 |
| | 100 | 43 | n.d.[f] | - | - | |
| | 180 | 5 days | 100 | 33 | n.d.[g] | |
| PLA, PCL, PET | 60 | 1 | 100 | - | - | 10.14272/reaction/SA-FUHFF-UHFFFADPSC-QVSUDYMNWS-UHFFFADPSC-NUHFF-NUHFF-NUHFF-ZZZ |
| | 100 | 20 | 100 | 24 | - | |
| | 180 | 28[h] | 100 | 39 | 100[i] | |
| PLA, PCL, PET | 180 | 4.5 | 100 | 18 | n.d.[j] | 10.14272/reaction/SA-FUHFF-UHFFFADPSC-QVSUDYMNWS-UHFFFADPSC-NUHFF-NUHFF-NUHFF-ZZZ.1 |
| PLA, PCL, PET[k] | 60 | 1 | 100 | - | - | 10.14272/reaction/SA-FUHFF-UHFFFADPSC-QVSUDYMNWS-UHFFFADPSC-NUHFF-NUHFF-NUHFF-ZZZ.5 |
| | 100 | 1 | - | 24 | - | |
| | 100 | 4.2[l] | - | 31 | - | |
| | 180 | 16.5 | - | 52 | n.d.[j] | |
| PLA, PCL, PET[k] | 60 | 1.6 | 100 | - | - | 10.14272/reaction/SA-FUHFF-UHFFFADPSC-QVSUDYMNWS-UHFFFADPSC-NUHFF-NUHFF-NUHFF-ZZZ.6 |
| | 100 | 1[m] | - | 15 | - | |
| | 100 | 2 | - | 12 | - | |
| | 180 | 5 | - | 19 | - | |
| | 180 | 7 | - | 21 | - | |
| | 180 | 22.5 | - | 41 | n.d.[j] | |
| PLA, PCL, PET[n] | 60 | 1 | 100 | - | - | 10.14272/reaction/SA-FUHFF-UHFFFADPSC-QVSUDYMNWS-UHFFFADPSC-NUHFF-NUHFF-NUHFF-ZZZ.2 |
| | 100 | 1 | - | 100 | - | |
| | 180[o] | 6 | - | 100 | 61 | |
| PLA, PCL, PET[n] | 60 | 1 | 100 | - | - | 10.14272/reaction/SA-FUHFF-UHFFFADPSC-QVSUDYMNWS-UHFFFADPSC-NUHFF-NUHFF-NUHFF-ZZZ.3 |
| | 100 | 1 | - | 100 | - | |
| | 180 | 5 | - | 100 | 60 | |

**a** Time at given temperature. **b** Determined with $^1$H NMR spectroscopic analysis and calculated according to literature.[13] **c** Determined with $^1$H NMR spectroscopic analysis and calculated according to literature.[16] **d** DMT was isolated from a clear reaction mixture indicating complete depolymerization of PET. **e** PLA powder was used. **f** $^1$H NMR spectroscopic analysis shows complete depolymerization of PLA to oligomers and MeLa. **g** DMT could not be isolated without traces of other depolymerization products, and the reaction mixture was turbid indicating incomplete depolymerization of PET. **h** Within the first 23 h, the reaction was stirred at 170 °C. After 1 h at 180 °C, MeOH (2 mL) was added to counter the loss of volatile components during the reaction. **i** Analyzed by $^1$H NMR spectroscopy ($Y_{DMT,\ isolated}$ = 36%). **j** DMT could not be isolated without traces of other depolymerization products, but a clear reaction mixture was obtained after the given reaction time. **k** MeLa and MeOH were removed after complete depolymerization was observed using $^1$H NMR spectroscopic analysis. 4 mL MeOH were added to the remaining polymer mixture. **l** After 1 h at 100 °C, $^1$H NRM analysis showed incomplete removal of MeLa was detected, thus the reaction time was prolonged to 4.2 h. **m** Due to incomplete removal of MeLa, the reaction was stopped after 1 h at 100 °C. All MeOH and remaining MeLa were removed *in vacuo*. 4 mL MeOH were added to the remaining polymer mixture. **n** MeLa and MeOH were removed after complete depolymerization was observed using $^1$H NMR spectroscopic analysis. 2 mol% $TMG_2e$ regarding the PCL ester bonds and 4 mL MeOH were added to the remaining polymer mixture. **o** Before heating the reaction to 180 °C, the reaction was stirred at 100 °C for 19 h.

Figure S17 shows the used PLA, PCL, and PET materials before depolymerization. PLA powder and PET powder were cryo-milled to 0.75 mm at HKI.

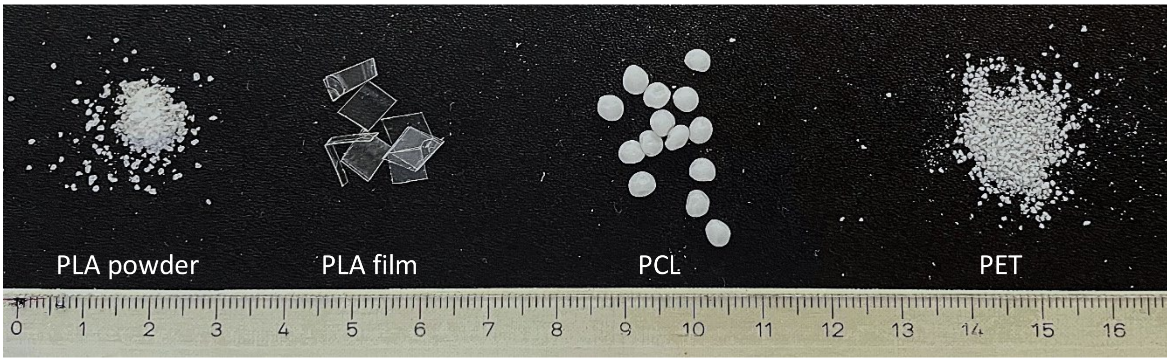


Figure S17. Used plastics before depolymerization: PLA powder (75 microns), PLA film (0.5x0.5 cm), PCL pellets (average Ø = 3 mm), and PET powder (75 microns).

In Figure S18, the transformation of the polymer material during the depolymerization of PLA film, PCL pellets, and PET powder is documented. First, all PLA film was dissolved and depolymerized (A), subsequently PCL-PET ovaloids (B) formed which transformed into one polymer lump (C). While increasing the $T$ to 80 °C, PCL was completely dissolved, and only PET powder was observable (D). At 100 °C, the PET powder started to get finer, and a homogenous milky suspension was obtained.

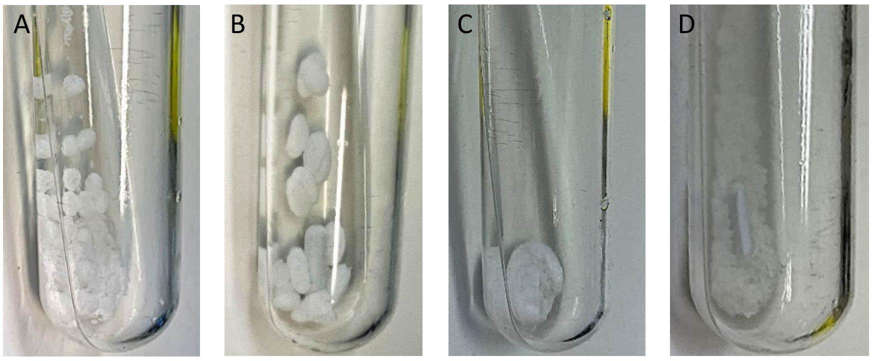


Figure S18. Visual transformation of plastics during the depolymerization of a plastic mix (PLA film, PCL pellets, PET powder).

### 8.1. In-depth evaluation of the performed (cascade) recycling experiments

Table 6 and Table S7 give an overview of the results obtained from multiple depolymerization experiments using five different polymer mixes: PLA(film)+PCL, PLA(film)+PET, PCL+PET, PLA(film)+PCL+PET (Figure S18), and PLA(powder)+PCL+PET.

The methanolysis of PLA and PET proceeded as indicated by previous experiments. PLA is depolymerized selectively to MeLa within 1 h at 60 °C under solvent-free conditions. The results for the methanolysis of PET in both binary polymer mixes are in good accordance with the results using only PET (Table 5). Within 5 h, a clear reaction mixture was obtained and DMT could be isolated in good yields ($Y_{DMT}$ = 73— 80%). In contrast to PLA and PET, the methanolysis of PCL was hindered by PLA, PET or their depolymerization products. In the presence of PET, PCL was depolymerized slower compared to the methanolysis of pure PCL reaching $Y_{MeHe}$ = 86% after 1 h and we could not observe complete methanolysis of PCL after 5 h at 180 °C. In the presence of MeLa obtained from the previous PLA depolymerization at 60 °C, a similar behavior was observed: At 100 °C, only 74% yield of MeHe could be determined after 1 h. A prolonged reaction time of 20 h only led to a slight increase of MeHe ($Y_{MeHe}$ = 79%). The methanolysis of PCL (397 mg, 3.48 mmol, 1.00 equiv) in the presence of MeLa (0.33 µL, 3.46 mmol, 0.995 equiv) lead to the complete inhibition of the PCL methanolysis (100 °C, $Y_{MeHe}$ = 0% after 1 h). We observed swelling of the polymer but only a small amount of PCL dissolved. Therefore, the lower yields might be due to the lower solubility of PCL under the given reaction conditions induced by the presence of MeLa. Further, we suggest that PET and/or DMT have a negative influence on the methanolysis of PCL as well. These results were further confirmed using mixes of PLA, PCL, and PET (Table 6 and Table S7).

Real plastic waste is not a homogeneous mixture and has polymers in different shapes, forms, and sizes, thus we investigated the influence of the physical form and size of the PLA particles on the depolymerization behavior of the plastic mixes. The use of PLA powder in the PLA+PCL+PET mix had a severe negative influence on the reaction. Due to the low solubility of the polymers in MeOH and the similar physical form of PLA and PET, a polymer lump was

formed immediately and we could not observe methanolysis products after a prolonged reaction time of 24 h at 60 °C. After increasing the temperature to 100 °C, PLA, PCL, and PET were depolymerized slowly. After 43 h, PLA was depolymerized completely to MeLa and oligomers. Resonances for MeHe and DMT could be identified using $^{1}$H NMR spectroscopic analysis. We monitored the reaction for five days but could not observe complete dissolution of all polymer particles. $^{1}$H NMR spectroscopic analysis showed complete methanolysis of the PLA oligomers to MeLa; dissolved PCL was depolymerized to MeHe ($Y_{\text{MeHe}}$ = 33%), and resonances for DMT and other depolymerization products of PET could be identified. Using PLA film, we observed complete selective methanolysis of PLA to MeLa within 1 h at 60 °C using $^{1}$H NMR spectroscopic analysis. At 60 °C, the PCL pellets and the PET powder formed first ovaloids and after 1 h the polymers formed a large bulk (Figure S18). Nonetheless, small resonances for PCL and MeHe were observed in the $^{1}$H NMR spectroscopic analysis. We increased the temperature to 100 °C to accelerate the depolymerization of PCL. At 80 °C, PCL was dissolved completely (Figure S18). After 20 h at 100 °C, $^{1}$H NMR spectroscopic analysis showed only slow methanolysis of PCL ($Y_{\text{MeHe}}$ = 24%) and traces of DMT in the reaction mixture indicating slow depolymerization of PET at low temperature. To enhance the depolymerization of PCL and PET the reaction temperature was increased to 180 °C. After 23 h at 170 °C and 28 h at 180 °C, a clear reaction mixture was obtained. PCL was depolymerized to 39% and PET to 100%. We observe improved depolymerization behavior using PLA film instead of PLA powder for PLA and PET. However, the conversion of PCL only increased by 6 percent points. Further, we conducted the experiment directly at 180 °C to simultaneously depolymerize all three polymers. After only 4.5 h, a clear solution was obtained suggesting complete depolymerization of PET. $^{1}$H NMR spectroscopic analysis showed complete methanolysis of PLA and PET. However, only a low MeHe yield could be determined ($Y_{\text{MeHe}}$ = 18%).

# 9. Depolymerization of commercially available PLLA cups

The PLLA cups were used for soft drinks, water, and warm coffee for several days (see Figure S19). For the depolymerization experiments, we cut the cups into small pieces without washing or drying. The obtained FAIR data for the experiment is available under doi: 10.14272/reaction/SA-FUHFF-UHFFFADPSC-LPEKGGXMPW-UHFFFADPSC-NUHFF-NUHFF-NUHFF-ZZZ.4.

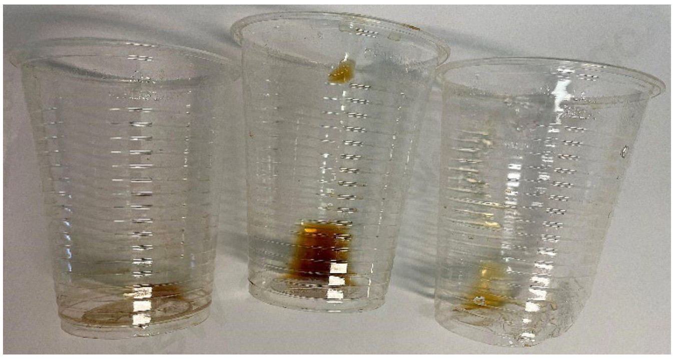

Figure S19. Used PLA cups from Ilip.

# 10. Post-consumer waste

## 10.1. Composition of post-consumer waste

Table S8 gives an overview of the composition of the post-consumer waste material received from AIMPLAS (Spain). It contains PET, polyethylene (PE), polyamides (PA), polystyrene (PS), polypropylene (PP), polyvinylchloride (PVC), and non-plastic material.

Table S8. Contents of post-consumer waste samples received from AIMPLAS.

| Waste sample | PET [%] | PE [%] | PA [%] | PS [%] | PP [%] | PVC [%] | Other (non-plastic material) [%] |
|---|---|---|---|---|---|---|---|
| Mixed plastic waste Spain | 0 | 89 | 4 | - | 3 | 4 | - |
| Mixed plastic waste (VFB) | 30 | 41 | - | 3 | 9 | - | 9 |
| PET bottles | 100 | - | - | - | - | - | - |
| PET trays | 87 | 12 | - | - | - | - | <1 |

The real waste materials were provided by AIMPLAS (Spain) and collected in Spain or Denmark (Vestforbrænding).

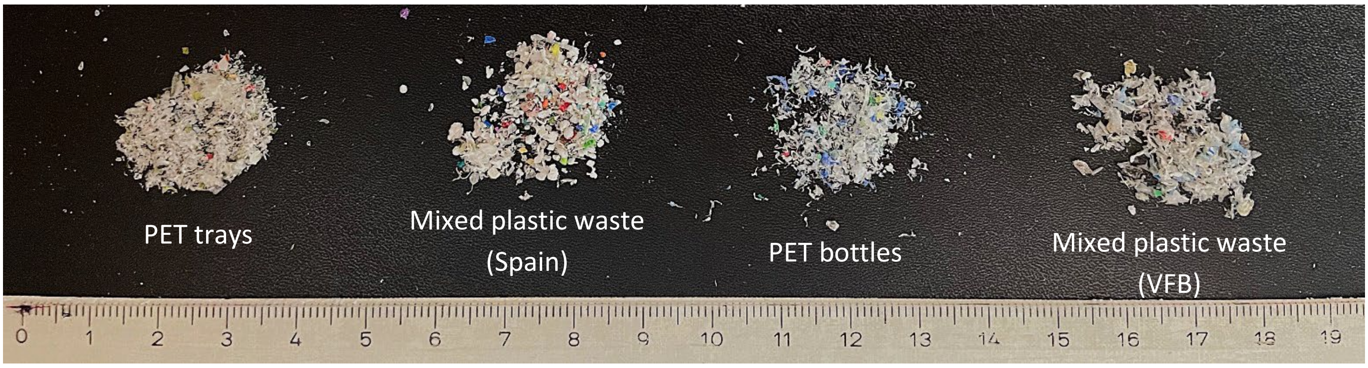


Figure S20. Different real waste material.

### 10.2. Depolymerization of post-consumer waste

The methanolysis and glycolysis of post-consumer PET waste was conducted and analyzed according to literature.[17-19] The obtained FAIR data for the experiments listed in Table 7 is available under the given dois in Table S9

Table S9. Link to the obtained FAIR data for the performed depolymerization experiments listed in Table 7.

| Waste sample | *m* [g] | $c_{cat}^{a}$ [mol%] | doi |
|---|---|---|---|
| Mixed plastic waste Spain | 1.0 | - | 10.14272/reaction/SA-FUHFF-UHFFFADPSC-QPKOBORKPH-UHFFFADPSC-NUHFF-NUHFF-NUHFF-ZZZ.7 |
| Mixed plastic waste (VFB)[c] | 2.25 | 2.0 | 10.14272/reaction/SA-FUHFF-UHFFFADPSC-WRSZSDHIFL-UHFFFADPSC-NUHFF-NUHFF-NUHFF-ZZZ |
| Mixed waste (VFB) | 1.0 | 2.6 | 10.14272/reaction/SA-FUHFF-UHFFFADPSC-QPKOBORKPH-UHFFFADPSC-NUHFF-NUHFF-NUHFF-ZZZ.8 |
| PET bottles | 1.0 | 0.8 | 10.14272/reaction/SA-FUHFF-UHFFFADPSC-QPKOBORKPH-UHFFFADPSC-NUHFF-NUHFF-NUHFF-ZZZ.5 |
| PET bottles | 0.67 | 1.0 | 10.14272/reaction/SA-FUHFF-UHFFFADPSC-QPKOBORKPH-UHFFFADPSC-NUHFF-NUHFF-NUHFF-ZZZ.3 |
| PET trays[b] | 0.67 | 2.3 | 10.14272/reaction/SA-FUHFF-UHFFFADPSC-WOZVHXUHUF-UHFFFADPSC-NUHFF-NUHFF-NUHFF-ZZZ.2 |
| PET trays | 1.0 | 0.9 | 10.14272/reaction/SA-FUHFF-UHFFFADPSC-QPKOBORKPH-UHFFFADPSC-NUHFF-NUHFF-NUHFF-ZZZ.6 |
| PET trays | 0.67 | 2.3 | 10.14272/reaction/SA-FUHFF-UHFFFADPSC-QPKOBORKPH-UHFFFADPSC-NUHFF-NUHFF-NUHFF-ZZZ.1 |
| PET trays | 3.0 | 1.2 | 10.14272/reaction/SA-FUHFF-UHFFFADPSC-QPKOBORKPH-UHFFFADPSC-NUHFF-NUHFF-NUHFF-ZZZ.2 |
| PET trays[20] | 7.0 | 1.1 | 10.14272/reaction/SA-FUHFF-UHFFFADPSC-QPKOBORKPH-UHFFFADPSC-NUHFF-NUHFF-NUHFF-ZZZ.9 |

**a** Regarding the PET repeating units. **b** MeOH (4 mL) was used. **c** MeOH (7 mL) was used.

# 11. Characteristics of used polymers

## 11.1. Differential scanning calorimetry

## 11.2. PLA film

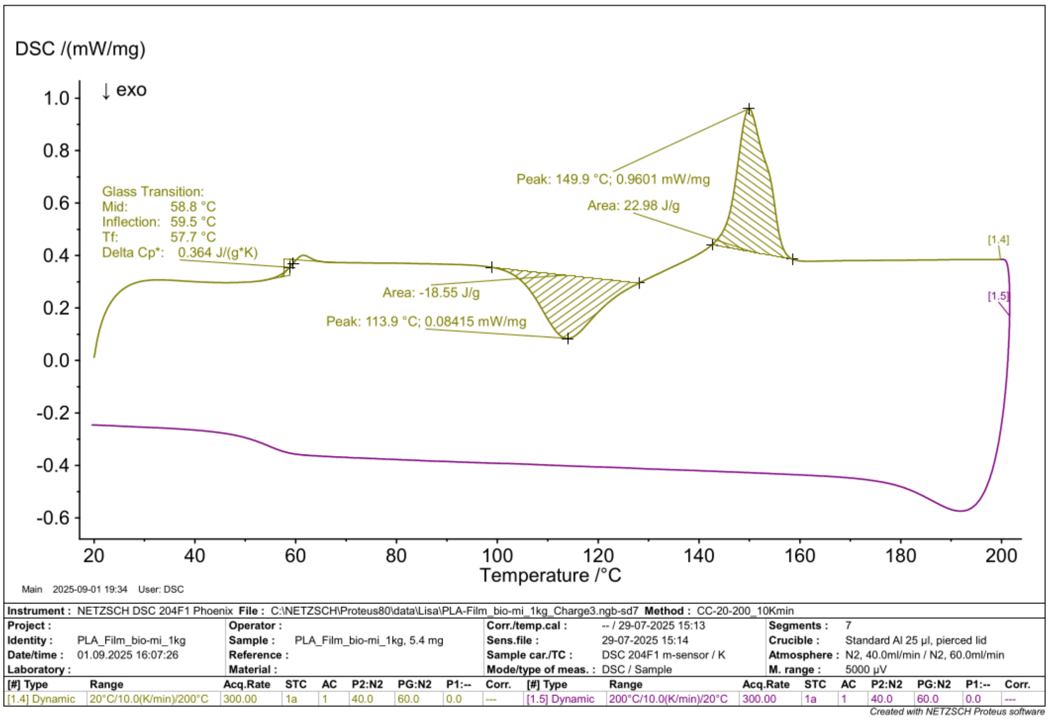


Figure 21. DSC of PLA film obtained from bio-mi Ltd. (Croatia).

## 11.3. PLA powder

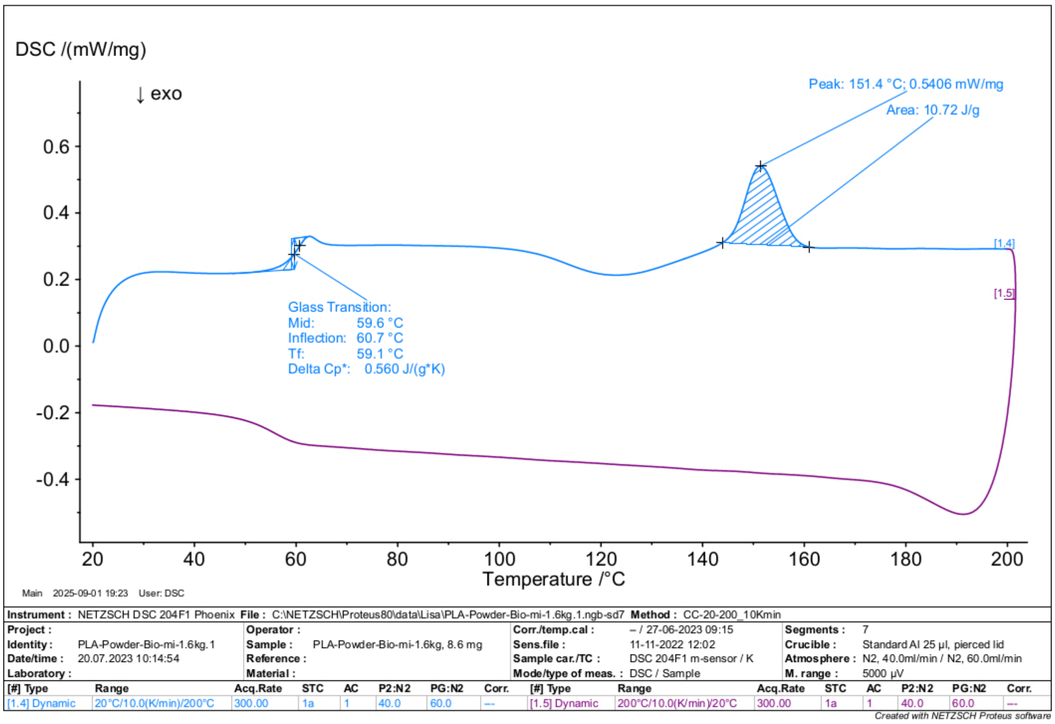


Figure 22. DSC of PLA film obtained from bio-mi Ltd. (Croatia).

## 11.4. PET powder

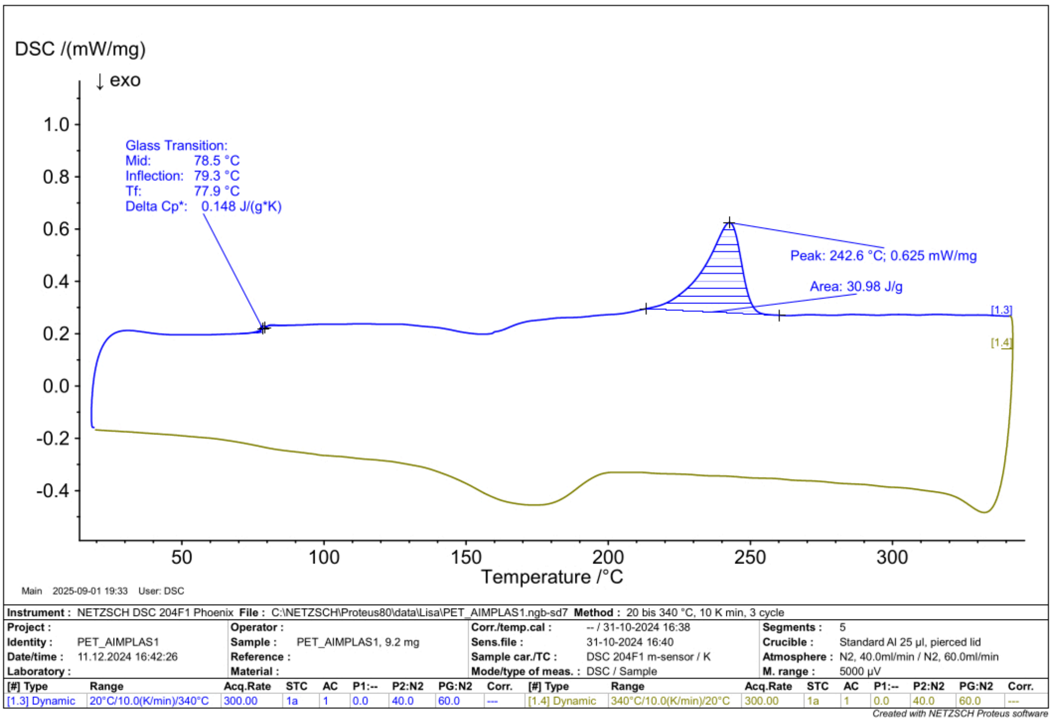


Figure 23. DSC of PET powder obtained from AIMPLAS (Spain).

## 11.5. Infrared spectroscopy

## 11.6. PLA film

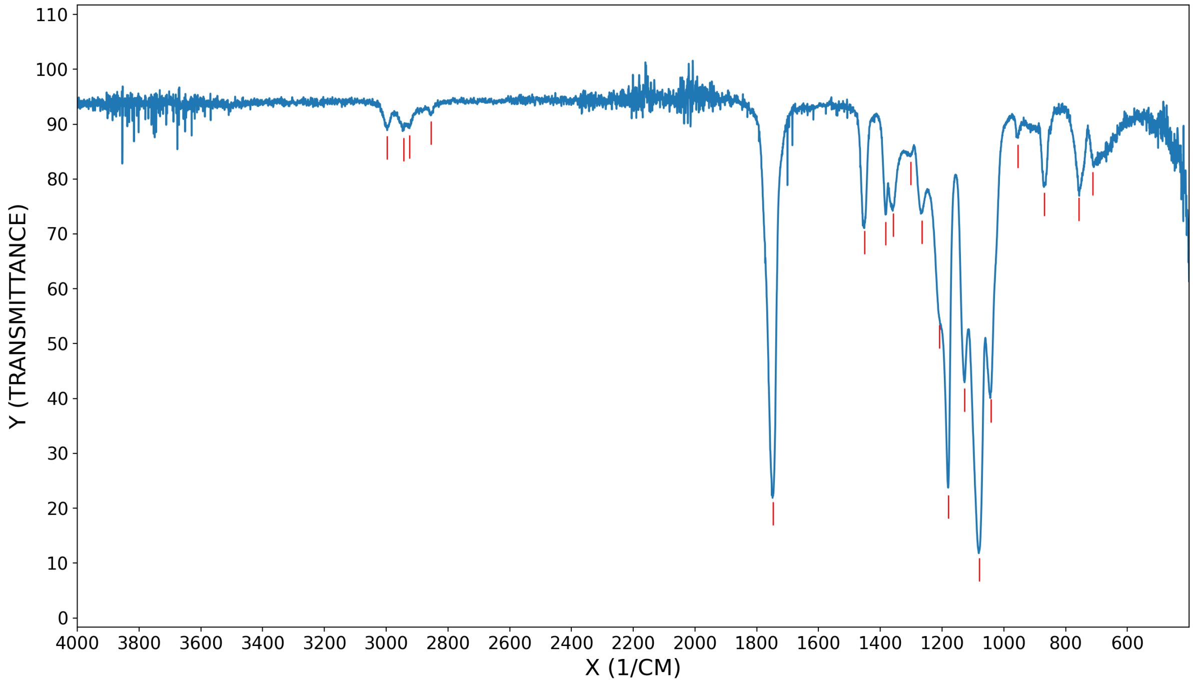


Figure 24. FTIR spectrum of PLA film as obtained from bio-mi Ltd. (Croatia).

**IR (ATR, ṽ)** = 2997 (vw), 2943 (vw), 2925 (vw), 2854 (vw), 1748 (vs), 1451 (w), 1383 (w), 1358 (w), 1302 (w), 1265 (w), 1209 (m), 1180 (vs), 1127 (s), 1079 (vs), 1042 (s), 955 (w), 869 (w), 757 (w), 712 (w) cm⁻¹.

### 11.7. PLA powder

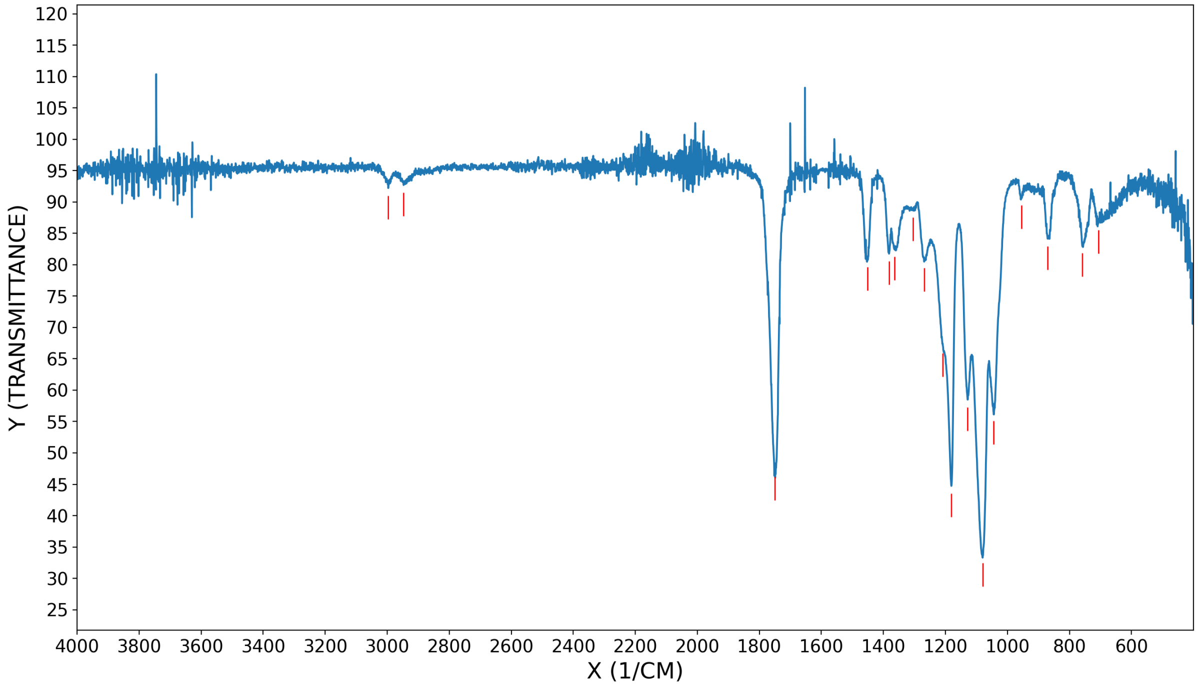


Figure 25. FTIR spectrum of PLA powder obtained from bio-mi Ltd. (Croatia) dissolved in THF.

**IR (ATR, ṽ)** = 2997 (w), 2947 (w), 1750 (vs), 1451 (w), 1382 (w), 1364 (w), 1305 (w), 1268 (w), 1208 (s), 1181 (vs), 1128 (s), 1080 (vs), 1044 (vs), 955 (w), 870 (w), 759 (w), 707 (w) $cm^{-1}$.

### 11.8. PLA cup

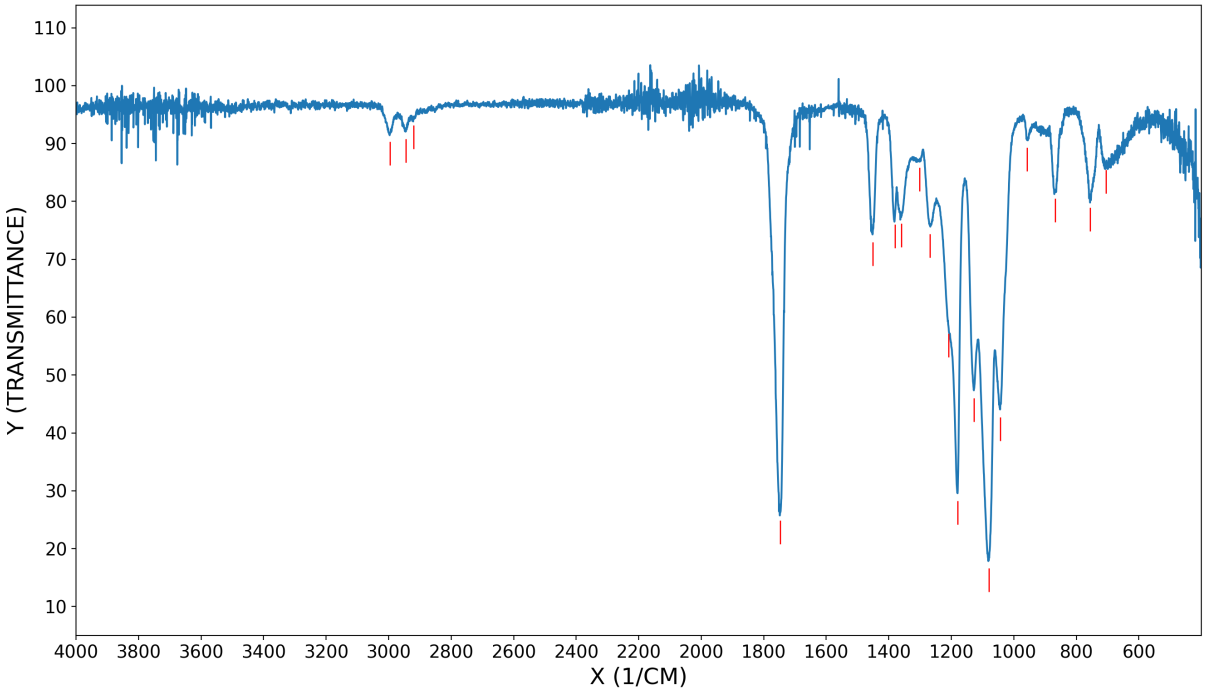


Figure 26. FTIR spectrum of PLA cup (Ilip Bio, Italy, product code: 61850 - LI20050TCE).

**IR (ATR, ṽ)** = 2996 (vw), 2945 (vw), 2920 (vw), 1748 (vs), 1452 (w), 1380 (w), 1360 (w), 1301 (w), 1268 (w), 1209 (m), 1180 (vs), 1128 (s), 1080 (vs), 1044 (s), 958 (w), 867 (w), 757 (w), 705 (w) $cm^{-1}$.

### 11.9. PCL pellets

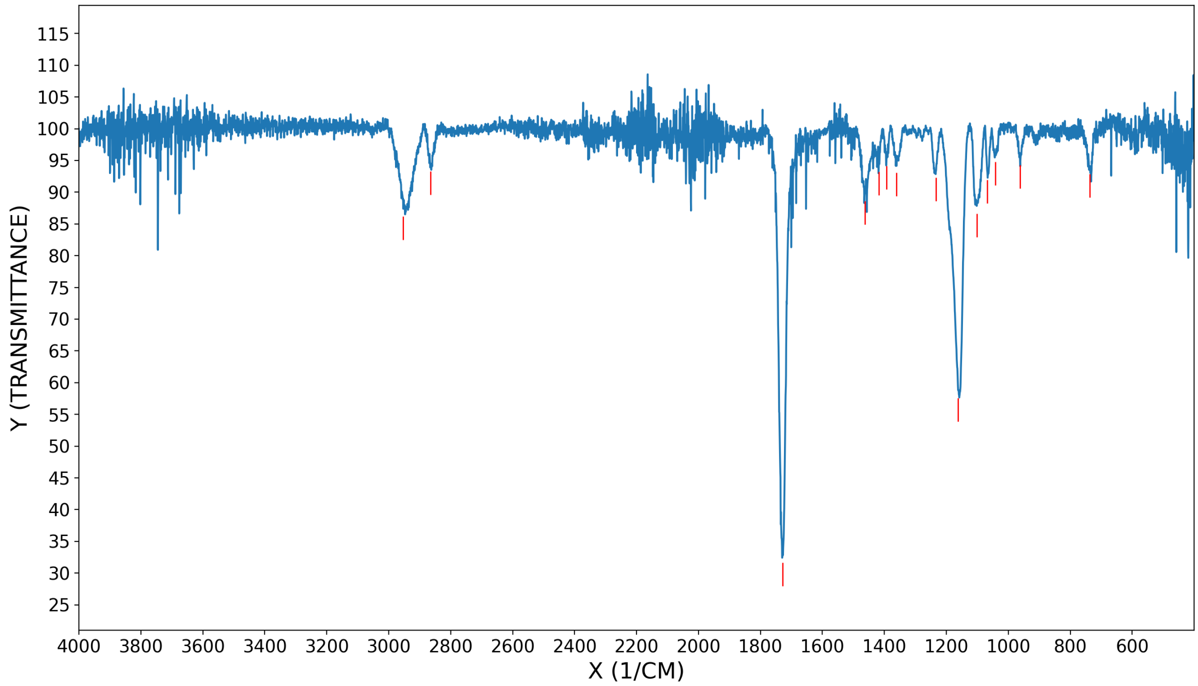


Figure 27. FTIR spectrum of PCL pellets purchased from Sigma Aldrich dissolved in THF.

**IR (ATR, ṽ)** = 2953 (w), 2865 (w), 1728 (vs), 1463 (w), 1418 (w), 1392 (w), 1361 (w), 1233 (w), 1162 (s), 1102 (w), 1068 (w), 1042 (w), 962 (w), 737 (w) $cm^{-1}$.

# 12. References


[1] H. E. Gottlieb, V. Kotlyar, A. Nudelman, "NMR Chemical Shifts of Common Laboratory Solvents as Trace Impurities" *J. Org. Chem.* **1997**, *62*, 7512.

[2] Mestrelab Research, 12.0.1 ed., Mestrelab Research, Santiago de Compostela, Spain, **2018**.

[3] Shimadzu, 2.15 ed., Shimadzu Corporation, Kyoto, Japan, **2016**.

[4] Y.-C. Huang, P. Tremouilhac, A. Nguyen, N. Jung, S. Bräse, "ChemSpectra: a web-based spectra editor for analytical data" *J. Cheminf.* **2021**, *13*, 8.

[5] H. Wittmann, A. Schorm, J. Sundermeyer, "Chelatliganden auf Basis peralkylierter Bis- und Tris-Guanidine" *Z. Anorg. Allg. Chem.* **2000**, *626*, 1583.

[6] O. Bienemann, R. Haase, U. Flörke, A. Döring, D. Kuckling, S. Herres-Pawlis, "Neue Bisguanidin-Kupfer-Komplexe und ihre Anwendung in der ATRP/ New Bisguanidine-Copper Complexes and their Application in ATRP" *Z. Naturforsch. B* **2010**, *65*, 798.

[7] G. B. Malle, Christian; Radisson, Xavier (L. O.-. D.I.P.I.), EP 1 532 964 A1, **2006**.

[8] S. Herres-Pawlis, A. Neuba, O. Seewald, T. Seshadri, H. Egold, U. Flörke, G. Henkel, "A Library of Peralkylated Bis-guanidine Ligands for Use in Biomimetic Coordination Chemistry" *Eur. J. Org. Chem.* **2005**, *2005*, 4879.

[9] H. Eilingsfeld, G. Neubauer, M. Seefelder, H. Weidincer, "Synthesen mit Amidchloriden, III. Synthese und Reaktionen von Chlorformamidiniumchloriden" *Chem. Ber.* **1964**, *97*, 1232.

[10] F. Santulli, M. Lamberti, M. Mazzeo, "A Single Catalyst for Promoting Reverse Processes: Synthesis and Chemical Degradation of Polylactide" *ChemSusChem* **2021**, *14*, 5470.

[11] L. A. Roman-Ramirez, P. McKeown, M. D. Jones, J. Wood, "Kinetics of Methyl Lactate Formation from the Transesterification of Polylactic Acid Catalyzed by Zn(II) Complexes" *ACS Omega* **2020**, *5*, 5556.

[12] L. Burkart, A. Eith, A. Hoffmann, S. Herres-Pawlis, "Open Loop Recycling - Guanidine Iron(II) Polymerization Catalyst for the Depolymerization of Polylactide" *Chem. Asian J.* **2023**, *18*, e202201195.

[13] P. McKeown, L. A. Roman-Ramirez, S. Bates, J. Wood, M. D. Jones, "Zinc Complexes for PLA Formation and Chemical Recycling: Towards a Circular Economy" *ChemSusChem* **2019**, *12*, 5233.

[14] M. Fuchs, M. Walbeck, E. Jagla, A. Hoffmann, S. Herres-Pawlis, "Guanidine Carboxy Zinc Complexes for the Chemical Recycling of Renewable Polyesters" *ChemPlusChem* **2022**, *87*, e202200029.

[15] C. Conrads, L. Burkart, S. Soerensen, S. Noichl, Y. Kara, J. Heck, A. Hoffmann, S. Herres-Pawlis, "Understanding structure-activity relationships: iron(II) complexes of "Legacy Guanidines" as catalysts for the synthesis of polylactide" *Catal. Sci. Technol.* **2023**, *13*, 6006.

[16] E. Cheung, C. Alberti, S. Bycinskij, S. Enthaler, "Zinc‐ Catalyzed Chemical Recycling of Poly(ϵ‐ caprolactone) Applying Transesterification Reactions" *ChemistrySelect* **2021**, *6*, 8063.

[17] M. Fuchs, P. M. Schafer, W. Wagner, I. Krumm, M. Walbeck, R. Dietrich, A. Hoffmann, S. Herres-Pawlis, "A Multitool for Circular Economy: Fast Ring-Opening Polymerization and Chemical Recycling of (Bio)polyesters Using a Single Aliphatic Guanidine Carboxy Zinc Catalyst" *ChemSusChem* **2023**, *16*, e202300192.

[18] T. Becker, A. Hermann, N. Saritas, A. Hoffmann, S. Herres-Pawlis, "Open- and Closed-Loop Recycling: Highly Active Zinc Bisguanidine Polymerization Catalyst for the Depolymerization of Polyesters" *ChemSusChem* **2024**, *17*, e202400933.

[19] J. Payne, M. Kamran, M. Davidson, M. D. Jones, "Versatile Chemical Recycling Strategies: Value-Added Chemicals from Polyester and Polycarbonate Waste" *ChemSusChem* **2022**, *15*, e202200255.

[20] V. Celestre, J. Madsen, C. Siracusa, L. Burkart, F. Quartinello, S. Herres-Pawlis, G. M. Guebitz, A. Pellis, A. E. Daugaard, "Microwave-Assisted Synthesis of Polyethylene Terephthalate as a Fast Screening Method for Catalyst Evaluation and for Evaluating Polymerizability of Monomers Recovered From Degraded Polyesters" *J. Appl. Polym. Sci.* **2025**, *142*, e57688.
